# SUMOylation inhibitors synergize with FXR agonists in combating liver fibrosis

**DOI:** 10.1038/s41467-019-14138-6

**Published:** 2020-01-13

**Authors:** Jiyu Zhou, Shuang Cui, Qingxian He, Yitong Guo, Xiaojie Pan, Pengfei Zhang, Ningning Huang, Chaoliang Ge, Guangji Wang, Frank J. Gonzalez, Hong Wang, Haiping Hao

**Affiliations:** 1 State Key Laboratory of Natural Medicines, Key Laboratory of Drug Metabolism and Pharmacokinetics, China Pharmaceutical University, 210009 Nanjing, China; 2 Department of Pharmacy, The First Affiliated Hospital of Anhui Medical University, 230022 Hefei, China; 3 Laboratory of Metabolism, Center for Cancer Research, National Cancer Institute, National Institutes of Health, Bethesda, MD 20892 USA

**Keywords:** Pharmacology, Liver fibrosis

## Abstract

Farnesoid X receptor (FXR) is a promising target for nonalcoholic steatohepatitis (NASH) and fibrosis. Although various FXR agonists have shown anti-fibrotic effects in diverse preclinical animal models, the response rate and efficacies in clinical trials were not optimum. Here we report that prophylactic but not therapeutic administration of obeticholic acid (OCA) prevents hepatic stellate cell (HSC) activation and fibrogenesis. Activated HSCs show limited response to OCA and other FXR agonists due to enhanced FXR SUMOylation. SUMOylation inhibitors rescue FXR signaling and thereby increasing the efficacy of OCA against HSC activation and fibrosis. FXR upregulates *Perilipin-1*, a direct target gene of FXR, to stabilize lipid droplets and thereby prevent HSC activation. Therapeutic coadministration of OCA and SUMOylation inhibitors drastically impedes liver fibrosis induced by CCl_4_, bile duct ligation, and more importantly NASH. In conclusion, we propose a promising therapeutic approach by combining SUMOylation inhibitors and FXR agonists for liver fibrosis.

## Abstract (web summary)

FXR agonists have been investigated for the treatment of non-alcoholic steatohepatitis and liver fibrosis but the clinical efficacy is not optimal. Here the authors show that enhanced FXR SUMOylation in activated hepatic stellate cells reduces FXR signaling and that this can be rescued by SUMOylation inhibitors.

## Introduction

Farnesoid X receptor (FXR, NR1H4) plays fundamental roles in maintaining the bile acid (BA) homeostasis^1^ and modulating lipids and glucose metabolism^2–5^, decreasing inflammation^6,7^, cell proliferation^8^, and cell death^9,10^. FXR has been exploited as a promising target for the treatment of various liver diseases^11^, including liver fibrosis^12^. FXR agonists with diverse chemical structures were developed^13–15^, notably obeticholic acid (OCA)^16^, a representative FXR agonist, that was approved for primary biliary cholangitis (PBC)^17,18^. More recently, OCA completed phase III clinical trial for nonalcoholic steatohepatitis (NASH) with fibrosis. In this trial, 18% of patients on 10 mg OCA and 23% on 25 mg OCA saw an improvement of liver fibrosis (≥1 stage), compared to 12% of placebo patients^19^. Although the phase III clinical trial of OCA for liver fibrosis with NASH reached one of the primary clinical endpoints, the responsive rate was not optimum^20^. In addition, OCA was not effective in decreasing PBC-induced liver fibrosis^21,22^. Moreover, FXR agonists cannot directly protect hepatocytes from death receptors engaged apoptosis^23^, which is a core pathological event in stimulating fibrotic development^24^. Additionally, FXR protein levels were reduced with the progress of fibrotic development and inflammation^23^. Except for the metabolic regulation and anti-inflammatory effects, the mechanism underlying how FXR agonists influence liver fibrosis remains largely unclear. A critical question is thus how to magnify the efficacies of FXR agonists in liver fibrosis based on intensive understanding of the molecular mechanisms.

Hepatic fibrosis is a scarring process of the liver that is characterized by increased extracellular matrix (ECM) resulting from chronic liver injury of any etiology, including bile acids accumulation, viral infection, alcoholic liver disease, and NASH^25,26^. Hepatic stellate cells (HSCs) play pivotal roles in the pathological development of liver fibrosis^27^. Under normal conditions, HSCs maintain a non-proliferative, quiescent phenotype with cytoplasmic lipid droplets (LDs) containing retinyl esters and triacylglycerols. Thus, HSCs were initially called Ito cells, lipocytes, and fat-storing cells^28^. Loss of cytoplasmic LDs is a key step in promoting HSC activation. LDs are coated by LD-associated proteins of the PAT domain family (perilipin, PLIN), including perilipin 1 (Plin1), adipophilin (ADRP, Plin2), tail-interacting protein of 47 kDa (TIP47, Plin3), S3-12 (Plin4), and OXPAT (Plin5)^29–32^. Loss of these proteins result in the degradation of LDs and activation of HSCs.

FXR is expressed in HSCs where it functions as a transcription factor regulating the expression of the small heterodimer partner (*Shp*) gene and microRNA-29a, and thereby reduces the expression of pro-fibrotic genes, including *Acta2* (encoding αSMA), transforming growth factor β1 (*Tgfb1*), collagen1a1 (*Col1a1*), *Col1a2*, tissue inhibitor of metalloprotease 1 (*Timp1*), and *Timp2*^33,34^. However, these results were questioned by later studies showing that cultured-activated HSCs were not responsive to FXR agonists; both the expression of *SHP* and *ACTA2* in culture-activated HSCs remained unaffected after 24 h of in vitro stimulation with 1 or 100 μM of OCA^35^.

We hypothesized that FXR may be dynamically regulated during the process of HSC activation and thus the responsiveness of HSCs to FXR agonists might differ between quiescent and activated status. As expected, quiescent but not activated HSCs are responsive to FXR agonists, and prophylactic but not therapeutic administration of OCA inhibits HSC activation and fibrosis development. Mechanistically, FXR SUMOylation is gradually enhanced in the process of HSCs activation, which compromises FXR signaling. *Plin1* is identified as a FXR target gene that is responsible for stabilizing LDs in HSCs. These data lead to a potential therapeutic approach to liver fibrosis by combining FXR agonists with SUMOylation inhibitors, which may provide insights into how to better harness FXR as a therapeutic target for the drug development of liver fibrosis induced by various etiologies.

## Results

### Prophylactic but not therapeutic OCA dosing impedes fibrosis

**Fig. 1:**
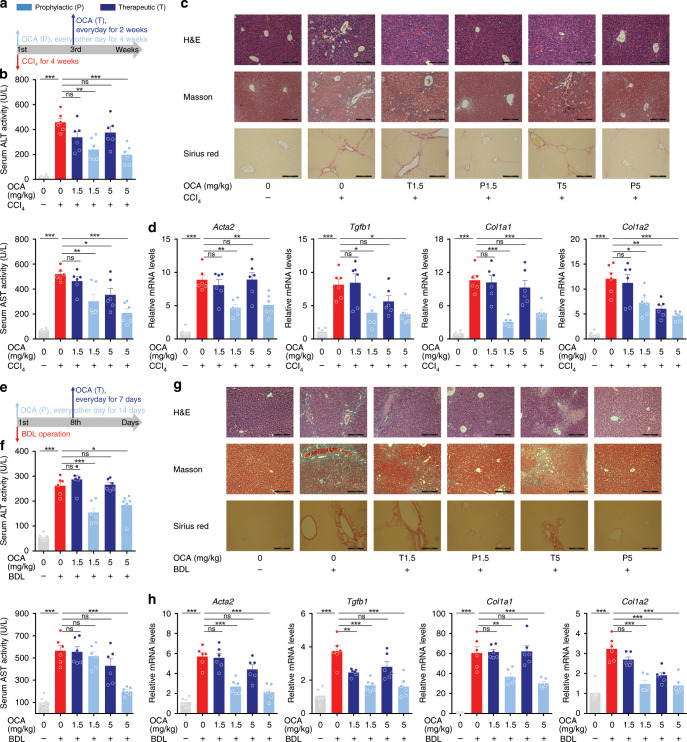
Prophylactic but not therapeutic administration of OCA alleviates liver fibrosis. The anti-fibrotic effects of prophylactic and therapeutic administration of OCA were evaluated in CCl_4_-induced **a**–**d** or BDL-induced **e**–**h** liver fibrosis models. **a** and **e** Mouse experiment procedure schemes. **b** and **f** Serum ALT and AST levels. **c** and **g** H&E, Masson and Sirius red staining of liver sections. Data are representative of *n* = 6 biological independent samples. Scale bar, 100 μm. **d** and **h** Levels of *Acta2*, *Tgfb1*, *Col1a1*, and *Col1a2* mRNAs in liver. *n* = 6 biologically independent samples within these experiments. Results are mean ± SEM, *P < 0.05, **P < 0.01, ***P < 0.001, and ns, statistically not significant, as assessed with ANOVA.

Previous studies on various animal models revealed that FXR agonists exert anti-fibrotic effects^36–39^, however, clinical trials revealed only modest efficacy in humans. Notably, OCA is not effective against liver fibrosis in PBC patients^21,22^ and only a quarter of NASH patients, despite statistical significance, showed improvement in liver fibrosis in a phase III clinical study^19^. Although there are diverse causes underlining the discrepancy between preclinical and clinical results, a big concern is that FXR agonists in most preclinical animal models were administered in a prophylactic manner, at a stage when there is no apparent fibrotic changes in the liver, which is totally different from the practical treatment of human patients. To address this concern, the effects of OCA were compared in liver fibrosis between prophylactic and therapeutic administration (Fig. 1a). As expected, prophylactic but not therapeutic administration of OCA significantly reduced serum ALT levels (Fig. 1b). Masson and Sirius red staining of liver section revealed a significant increase in the fibrotic surface upon CCl_4_ treatment. Compared with the CCl_4_-treated group, the prophylactic arm showed marked reduction in fibrotic surface, while the therapeutic arm showed marginal reduction (Fig. 1c). In line with the histological analysis, results from the mRNA expression of pro-fibrotic genes (including *Acta2*, *Col1a1*, *Col1a2*, and *Tgfb1*) further demonstrated that prophylactic but not therapeutic administration of OCA were effective against liver fibrosis (Fig. 1d). Consistently, mRNA levels of various pro-fibrotic genes in HSCs isolated from the mice with prophylactic but not therapeutic treatment of OCA showed dramatic reduction (Supplementary Fig. 1). Since loss of LDs is a hallmark of HSC activation, the lipid contents in HSCs were measured. The contents of retinoic acid (RA), triglycerides (TG), cholesterol (CHO) in HSCs from CCl_4_-treated mice were all significantly reduced, and prophylactic but not therapeutic treatment of OCA reversed the loss of these lipids (Supplementary Fig. 1). These findings were validated in BDL-induced liver fibrosis (Fig. 1e). In line with the results in CCl_4_-treated mice, prophylactic but not therapeutic administration of OCA showed anti-fibrotic effects in BDL mice, as revealed by serum ALT and AST levels (Fig. 1f), histological analysis (Fig. 1g), as well as the expression of pro-fibrotic genes (Fig. 1h). Results from primary HSCs also demonstrated that prophylactic but not therapeutic administration of OCA prevented HSC activation and LD loss (Supplementary Fig. 1).

**Fig. 2:**
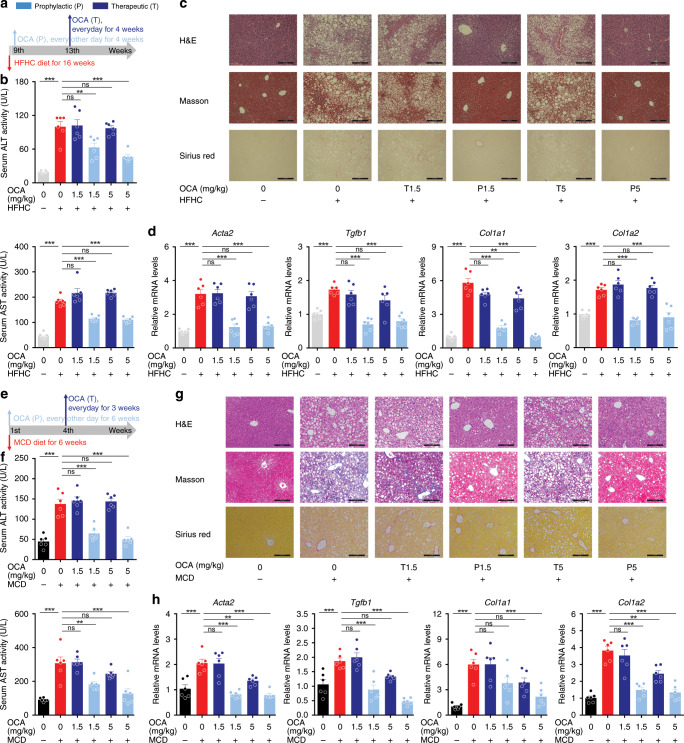
Prophylactic but not therapeutic OCA administration cures NASH-induced fibrosis. The effects of prophylactic and therapeutic administration of OCA were evaluated in NASH-induced fibrosis models caused by HFHC **a**–**d** and MCD **e**–**h** diet. **a** and **e** Mouse experiment procedure schemes. **b** and **f** Serum ALT and AST levels. **c** and **g** H&E, Masson and Sirius red staining of liver sections. Data are representative of *n* = 6 biological independent samples. Scale bar, 100 μm. **d** and **h** Levels of *Acta2*, *Tgfb1*, *Col1a1*, and *Col1a2* mRNAs in liver. *n* = 6 biologically independent samples within these experiments. Results are mean ± SEM, *P < 0.05, **P < 0.01, ***P < 0.001, and ns, statistically not significant, as assessed with ANOVA.

NASH is a major pathological driver of liver fibrosis. We thus further tested this concept in two typical NASH models. Mice were fed with high fat plus high CHO diet and fructose/sucrose water (HFHC) for 16 weeks (Fig. 2a). HFHC mice were characterized with increased serum ALT and AST levels, pronounced steatosis, inflammation, ballooning, and fibrosis, and enhanced expression of pro-fibrotic genes (Fig. 2b–d). Prophylactic (from the 9th week) but not therapeutic (from the 13th week) administration of OCA significantly reduced serum ALT and AST levels (Fig. 2b), and ameliorated liver fibrosis (Fig. 2c, d). Consistently, fibrotic gene expressions and the contents of RA, TG, and CHO in isolated HSCs were reduced by prophylactic but not therapeutic treatment of OCA (Supplementary Fig. 2). Similar results were observed in methionine and choline-deficient diet (MCD)-induced NASH model (Fig. 2e–h, Supplementary Fig. 2). These results indicate that the results collected from prophylactic dosing of OCA that is widely applied in preclinical studies might not be suitable for direct translation to the clinic.

### OCA is effective in quiescent but not activated HSCs

**Fig. 3:**
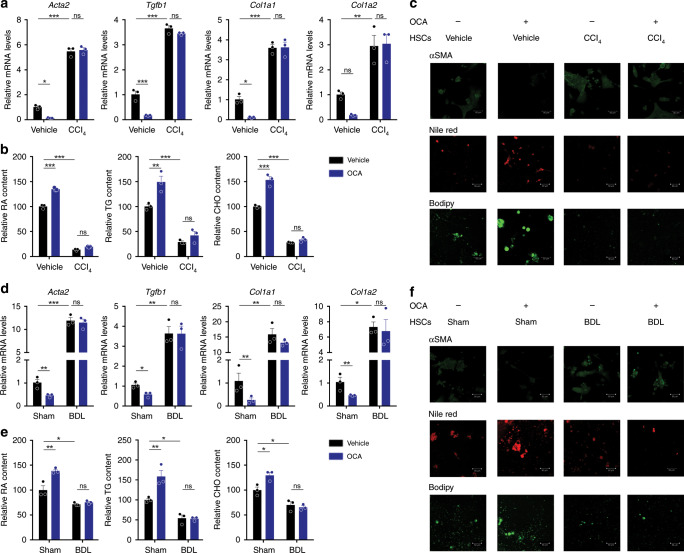
OCA is ineffective against already activated HSCs. Effect of OCA on HSC activation were compared between quiescent and activated HSCs, caused by CCl_4_ treatment **a**–**c** or BDL operation **d**–**f**. Freshly isolated HSCs were treated with OCA starting 1 day after seeding and collected for analysis after 4 days in culture. **a** and **d** mRNA levels of *Acta2*, *Col1a1*, *Col1a2*, and *Tgfb1* mRNAs in primary HSCs. **b** and **e** Lipids quantitation. **c** and **f** αSMA, Bodipy and Nile red staining of HSCs, data are representative of *n* = 3 biological independent samples. Scale bar, 50 μm. *n* = 3 biologically independent samples within these experiments. Results are mean ± SEM, *P < 0.05, **P < 0.01, ***P < 0.001, and ns, statistically not significant, as assessed with ANOVA.

Because HSC activation is a hallmark of liver fibrosis, we hypothesized that the differentiated responses of quiescent and activated HSCs to FXR agonists may underlie the discrepancy between prophylactic and therapeutic treatments. To this end, primary HSCs were isolated from vehicle-treated or CCl_4_-treated mice and cultured in vitro to promote their auto-activation. Significant inhibition of pro-fibrotic gene transcription upon OCA treatment was observed in primary HSCs from vehicle-treated mice, while marginal inhibition was observed in those from CCl_4_-treated mice (Fig. 3a). OCA prevented LD loss in HSCs from vehicle-treated mice as revealed by analysis of RA, TG, and CHO, and the lipid staining with Bodipy and Nile red, while no obvious effect was observed in cells isolated from CCl_4_-treated rats (Fig. 3b, c). Similar results were obtained from the study of HSCs of BDL mice. OCA reduced the mRNA levels of pro-fibrotic genes and prevented HSC LD loss from sham-operated mice but not BDL mice (Fig. 3d–f). Consistently, marginal inhibition in pro-fibrotic gene expression as revealed by mRNA levels after OCA treatment was observed in HSC-T6 cells with facilitated activation by TGFβ1 (Supplementary Fig. 3). Together, these results indicate that OCA is effective in quiescent but not pre-activated HSCs.

### SUMOylation underlies reduced FXR activity in activated HSCs

**Fig. 4:**
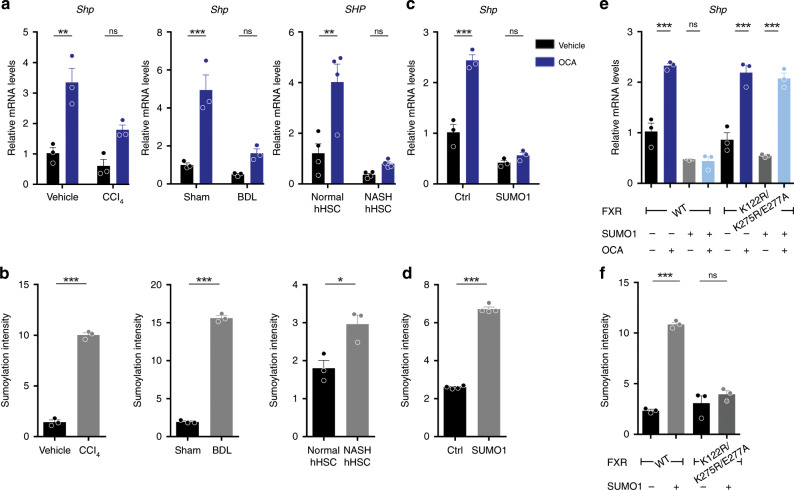
Elevated SUMOylation of FXR in activated HSCs represses its transcriptional activity. **a** OCA failed to induce the expression of SHP in activated HSCs, caused by CCl_4_ treatment, BDL operation, and from NASH patients. **b** SUMOylation of FXR elevated in activated HSCs, caused by CCl_4_ treatment, BDL operation, and from NASH patients, as analyzed by Protein SUMOylation Assay Ultra Kit. **c**, **d** *Shp* mRNA levels **c** and FXR SUMOylation **d** in cells transfected with SUMO1 plasmid. **e**, **f** *Shp* levels **e** and FXR SUMOylation **f** in cells transfected with WT or SUMO-site mutant FXR plasmid together with SUMO1 plasmid (*n* = 3 biologically independent samples within these experiments). Results are mean ± SEM, *P < 0.05, **P < 0.01, ***P < 0.001, and ns, statistically not significant, as assessed with Student’s *t*-test or ANOVA.

Because FXR is a transcriptional factor, we reasoned that its transactivity might be compromised in activated HSCs. The FXR target gene *Shp* mRNA expression in HSCs from healthy mice was significantly increased after OCA administration, while its induction by OCA was increased but attenuated in CCl_4_-treated or BDL-treated mice (Fig. 4a). Similar results were obtained from the analysis of other FXR agonists, including GW4064 and WAY-362450 in HSC-T6 cells treated with vehicle or TGFβ1 (Supplementary Fig. 4). In addition, primary human HSCs from healthy donors, were more responsive to OCA stimulation as compared to HSCs from NASH patients (Fig. 4a). These results strongly support that the function of FXR is gradually lost in the process of HSCs activation. We first asked whether the protein levels of FXR in HSCs are reduced as found in hepatocytes^23^. Surprisingly, the mRNA and protein levels of FXR remained nearly unchanged during the activation of HSCs (Supplementary Fig. 5a, b).

Since transcriptional activities of NRs may also be modulated by post translational modifications (PTMs)^40,41^, the PTMs of FXR were explored. Results from Co-IP assay showed that FXR SUMOylation was gradually enhanced during the activation of primary HSCs, phosphorylation of FXR was suppressed in highly activated HSCs, while acetylation of FXR remained unchanged (Supplementary Fig. 5c). The molecular masses of FXR and SUMO1 are about 55 and 15 kDa, respectively, and thus the SUMOylated FXR would be detected at about 70 kDa by both anti-FXR and anti-SUMO1 antibodies. Western blot assays were conducted to detect the SUMOylated form of FXR protein. In accordance with the results from Co-IP, SUMOylated FXR was obviously elevated in HSCs from CCl_4_-treaed and BDL-operated mice than that in HSCs from control mice (Supplementary Fig. 5d). The elevation of FXR SUMOylation in activated HSCs was further validated by use of a SUMOylation ELISA kit (Supplementary Fig. 5e and Fig. 4b). Notably, the SUMOylation of FXR was found significantly higher in primary human HSCs from NASH patients compared to healthy donors (Fig. 4b). Mammals express three SUMO proteins that can be divided into two families, SUMO1 and closely related SUMO2/3^41,42^. In vitro SUMOylation assay demonstrated that both SUMO1 and SUMO2/3 could be attached to recombinant FXR-GFP protein (Supplementary Fig. 5f), in line with previous reports^43,44^. Overexpression of either SUMO1 or SUMO2 in HSCs significantly enhanced FXR SUMOylation. However, knock-down of *Sumo1* but not *Sumo*2 in HSCs reduced FXR SUMOylation (Supplementary Fig. 5g–j). Furthermore, SUMO1 overexpression resulted in not only increased FXR SUMOylation, but also reduced response to OCA treatment, as demonstrated by analysis of *Shp* mRNA levels (Fig. 4c, d, Supplementary Fig. 5k). Lys^122^, Lys^275^, and Glu^277^ of FXR had been previously identified as the SUMO consensus sites^43^. In line with previous reports, single mutation of K122R, K275R, or E277A reduced FXR SUMOylation, while triple mutations of these sites almost completely abolished SUMO conjugation (Supplementary Fig. 5l). Analysis of the transcriptional activity of these mutants by *Shp* expression also demonstrated that SUMOylation at Lys^122^, Lys^275^, and Glu^277^ of FXR drastically repressed its transactivity (Supplementary Fig. 5m). The triple mutant form of FXR was resistant to SUMOylation-caused loss in transcriptional activity in primary HSCs (Fig. 4e, f, Supplementary Fig. 5n). Together, these results strongly support that SUMOylation is a pivotal factor regulating FXR transactivity.

### SUMOylation inhibitor restores FXR activity and function

**Fig. 5:**
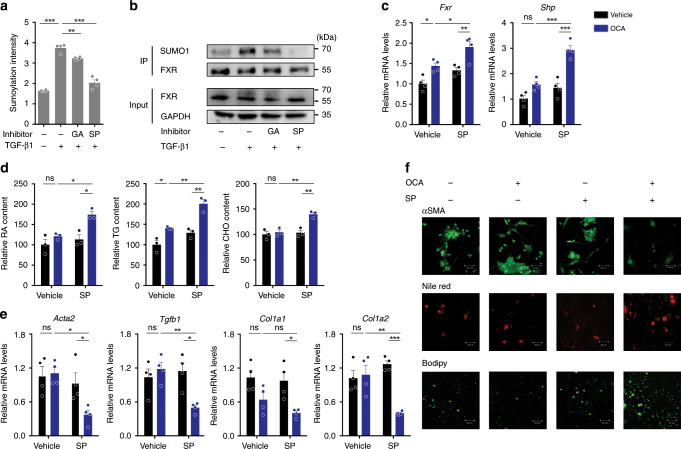
SUMOylation inhibition restores FXR transactivity and inhibits HSC activation. **a**, **b** SP and GA significantly inhibit FXR SUMOylation in activated HSCs by Protein SUMOylation Assay Ultra Kit (*n* = 4 biologically independent samples) **a** and Co-IP assay (representative of *n* = 3 biologically independent samples) **b**. **c** SP treatment restored the transcriptional activity of FXR. Activated HSCs were treated with OCA in the presence or absence of SP. *Fxr* and *Shp* mRNA levels (*n* = 4 biologically independent samples). **d**–**f** SP treatment restored the benefits of OCA in precluding HSC activation. Activated HSCs were treated with OCA in the presence or absence of SP. Lipid quantitation analysis (*n* = 3 biologically independent samples) **d**, pro-fibrotic gene mRNA levels (*n* = 4 biologically independent samples) **e**, and αSMA, Bodipy, and Nile red staining of HSCs (representative of *n* = 3 biologically independent samples. Scale bar, 50 μm). **f** Results are mean ± SEM, *P < 0.05, **P < 0.01, ***P < 0.001, and ns, statistically not significant, as assessed with ANOVA.

Because SUMOylation determines FXR transactivation, we supposed that a SUMOylation inhibitor would synergize with FXR agonists in suppressing HSC activation. To this end, a panel of SUMOylation inhibitors were screened. Results from SUMOylation ELISA kit assays, Co-IP assays, and western blot assays demonstrated that both GA and SP could significantly inhibit SUMOylation of FXR (Fig. 5a, b, Supplementary Fig. 5o). OCA efficiently upregulated the FXR target gene *Shp* in the presence of SP (Fig. 5c). SP treatment also restored FXR transactivation in activated HSCs when exposed to other FXR agonists including GW4064 and WAY-362450 (Supplementary Fig. 6). Additionally, treatment with GA, another SUMOylation inhibitor, also restored FXR transactivation in activated HSCs (Supplementary Fig. 6).

We next tested whether SUMOylation inhibitors could restore FXR function of inhibiting HSC activation. Primary HSCs were isolated and cultured for 4 days in the presence or absence of SP. In culture-activated HSCs, OCA alone was insufficient in increasing the storage of lipids and decreasing the pro-fibrotic biomarkers (Fig. 5d–f). In contrast, a combination of OCA and SP increased the lipid storage and decreased all pro-fibrotic biomarkers (Fig. 5d–f). The enhanced effects against HSC activation were also observed for other FXR agonists combined with SP, as well as the combination of GA and OCA (Supplementary Fig. 6c). Together, these results support that SUMOylation inhibition is capable of restoring FXR activity in activated HSCs and thereby inhibiting the fibrotic change of HSCs.

### SUMOylation inhibitor synergizes with OCA against fibrosis

**Fig. 6:**
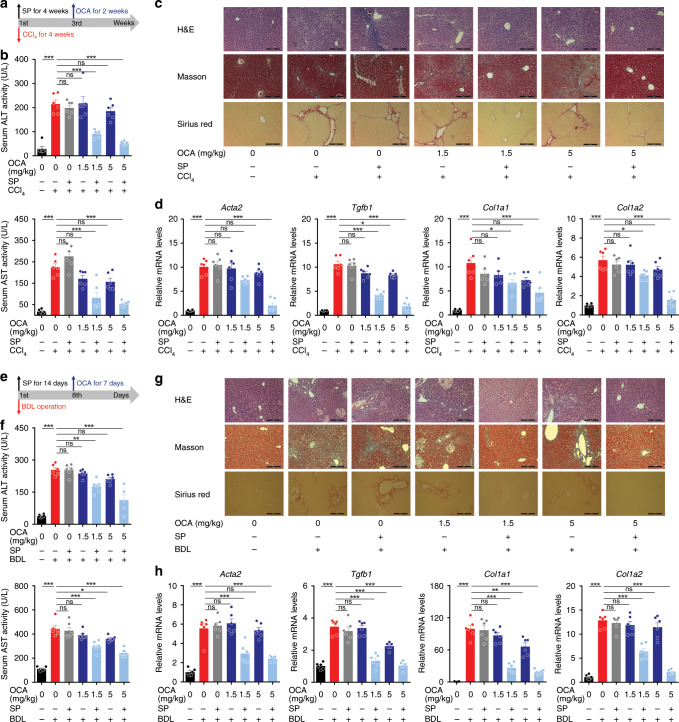
SUMOylation inhibition restores the anti-fibrotic activity of OCA. Fibrotic mice, developed by CCl_4_ treatment **a**–**d** or BDL operation **e**–**h**, were treated with OCA in the presence or absence of SP. *n* = 6 biologically independent samples within these experiments. **a** and **e** Mouse experiment procedure schemes. **b** and **f** Serum ALT and AST levels. **c** and **g** H&E, Masson, and Sirius red staining of liver sections (representative of *n* = 6 biologically independent samples. Scale bar, 100 μm). **d** and **h** mRNA expression of pro-fibrotic genes. Results are mean ± SEM, *P < 0.05, **P < 0.01, ***P < 0.001, and ns, statistically not significant, as assessed with ANOVA.

Since SUMOylation inhibitors synergize with FXR agonists in inhibiting HSCs activation, these inhibitors may also potentiate the therapeutic FXR agonist decrease of liver fibrosis. Mice were injected with SP upon after CCl_4_, and then treated with OCA two weeks later (Fig. 6a). Serum ALT and AST levels were dramatically reduced upon OCA treatment together with SP (Fig. 6b), but not by OCA or SP alone. Histological analysis also demonstrated that co-administration of OCA and SP reduced ECM accumulation and fibrosis development (Fig. 6c). Consistently, the mRNA levels from pro-fibrotic genes were all reduced upon combined treatment with OCA and SP (Fig. 6d). Primary HSCs were isolated to further validate the anti-fibrotic effects of this combination. As expected, SP treatment significantly inhibited FXR SUMOylation in HSCs (Supplementary Fig. 7). In the presence of SP, OCA significantly down-regulated the mRNA levels of pro-fibrotic genes, up-regulated *Shp* mRNA, and prevented LD loss (Supplementary Fig. 7). Similar results were obtained from BDL-induced fibrosis model. SP treatment significantly synergized with OCA in attenuating liver fibrosis as demonstrated by serum aminotransferases, histological analysis, and the mRNA levels of pro-fibrotic genes (Fig. 6e–h). Moreover, in freshly isolated HSCs, a combination of SP and OCA strongly down-regulated mRNA levels of pro-fibrotic genes, up-regulated *Shp* mRNA and prevented LD loss (Supplementary Fig. 7).

**Fig. 7:**
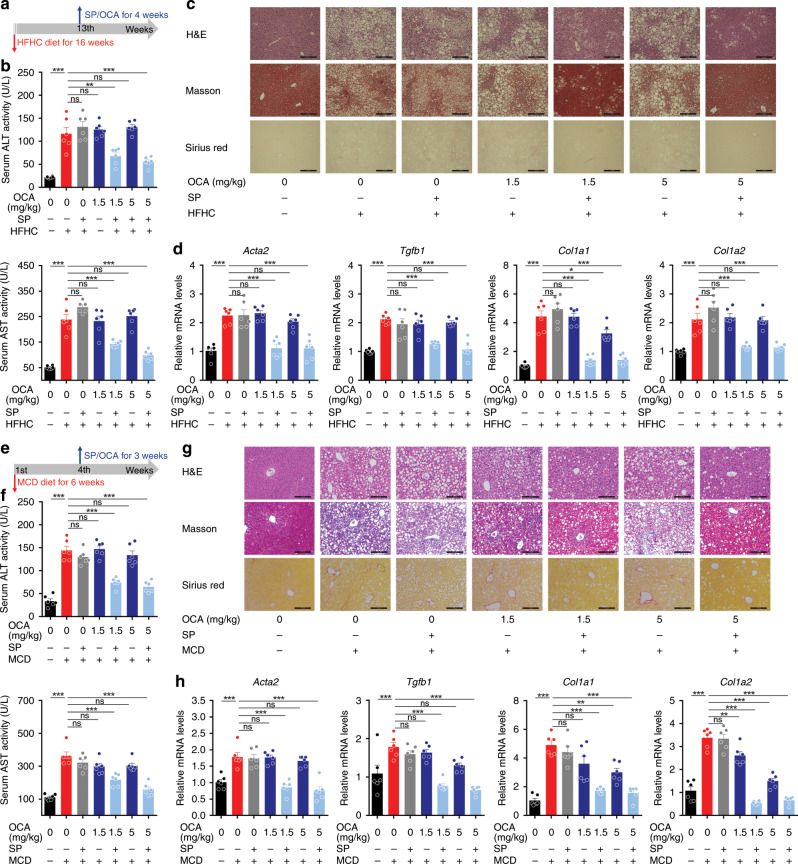
SUMOylation inhibition restores the anti-fibrotic activity of OCA in NASH models. Mice were fed with HFHC **a**–**d** or MCD **e**–**h** diet to induce NASH-associated fibrosis and treated with OCA in the presence or absence of SP. *n* = 6 biologically independent samples within these experiments. **a** and **e** Mouse experiment procedure schemes. **b** and **f** Serum ALT and AST levels. **c** and **g** H&E, Masson, and Sirius red staining of liver sections (representative of *n* = 6 biologically independent samples. Scale bar, 100 μm). **d** and **h** mRNA expression of pro-fibrotic genes. Results are mean ± SEM, *P < 0.05, **P < 0.01, ***P < 0.001, and ns, statistically not significant, as assessed with ANOVA.

In the clinic, NASH is a pivotal pathological cause in promoting liver fibrosis and a panel of FXR agonists has been developing for NASH fibrosis. We thus further validate the effects of SP and OCA combination against hepatic fibrosis in NASH models induced by HFHC diet as well as MCD diet. Individual administration of OCA showed marginal effects on serum aminotransferase levels, pathological improvement, and fibrotic gene expressions. In contrast, when combined with SP, OCA significantly reduced serum levels of aminotransferases and improved liver histological features including steatosis, inflammatory infiltration, and ballooning (Fig. 7b, c). Moreover, the combination of OCA and SP reduced ECM accumulation and mRNA levels of pro-fibrotic genes (Fig. 7c, d). In agreement, this combination also reduced mRNA levels of pro-fibrotic genes and restored lipid contents in primary HSCs (Supplementary Fig. 8). As expected, combined OCA and SP treatment also significantly impeded fibrotic development in MCD-induced NASH model (Fig. 7f–h, Supplementary Fig. 8). These results collectively support the view that a combination of SUMOylation inhibitors and FXR agonists could be a promising therapeutic approach to treat liver fibrosis caused by toxin, cholestasis, and NASH.

### FXR agonists stabilize lipid droplet **via** regulating Plin1

We next asked how SUMOylation inhibition, via restoration of FXR function, can synergize with FXR agonists in inhibiting HSCs activation and decreasing fibrosis. HSCs activation was associated with decreased lipid accumulation (Supplementary Fig. 9)^45^, and thus FXR agonists might inhibit HSCs activation via stabilizing LD. Cultured HSC-T6 cells were loaded with ROH and FAs to promote lipid accumulation and LD formation. As expected, cells loaded with lipids showed decreased αSMA staining and mRNA levels of pro-fibrotic genes (Supplementary Fig. 9), indicating that preventing LD loss may contribute to inhibiting HSC activation. In culture-activated primary HSCs, OCA pre-treatment was able to prevent LD loss (Supplementary Fig. 10). Since LD degradation is largely caused by the loss of LD-associated proteins^46,47^, the expression profiles of those proteins in culture-activated HSCs was analyzed. Surveying mRNA expression of Plin family members revealed that the expression of *Plin1*, but not other *Plins*, could be up-regulated by OCA, as well as two other FXR agonists (Supplementary Fig. 10). Primary HSCs from *Fxr*^−/−^ mice, in comparison with that from WT mice, were characterized with reduced mRNA levels of both *Shp* and *Plin1*, enhanced levels of various pro-fibrotic genes, and decreased lipid content (Supplementary Fig. 10).

**Fig. 8:**
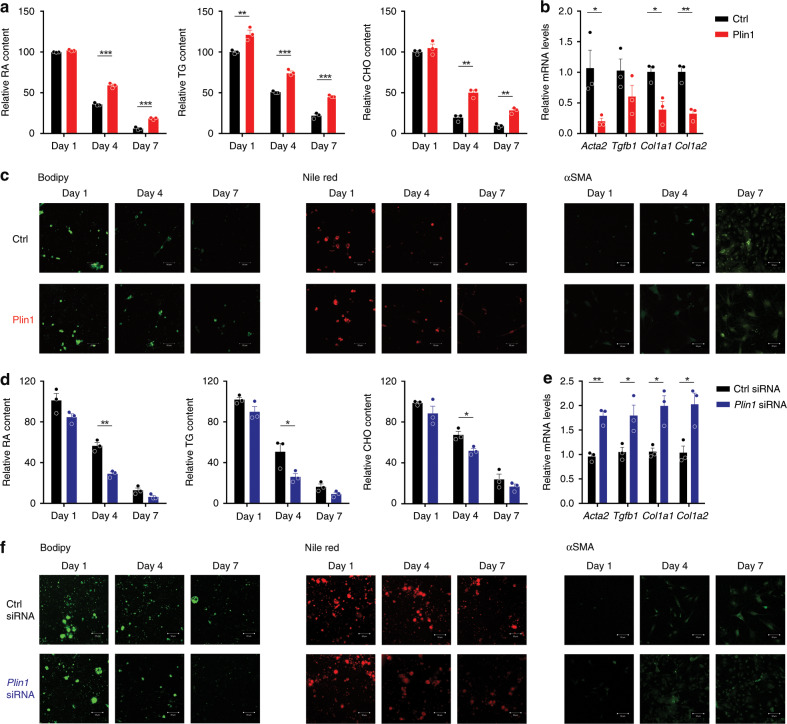
LD stabilization by Plin1 prevents HSC activation. **a**–**c** Plin1 overexpression precluded LD loss and HSC activation (*n* = 3 biologically independent samples within these experiments). **a** Lipids quantitation. **b** mRNA expression of pro-fibrotic genes. **c** Bodipy, Nile red, and α-SMA staining of cells (representative of *n* = 3 biologically independent samples). **d**–**f** Plin1 knock-down aggravated LD loss and HSC activation (*n* = 3 biologically independent samples within these experiments). **d** Lipids quantitation. **e** Pro-fibrotic gene mRNA levels. **f** Bodipy, Nile red, and α-SMA staining of cells (representative of *n* = 3 biologically independent samples. Scale bar, 50 μm). Results are mean ± SEM, *P < 0.05, **P < 0.01, as assessed with Student’s *t*-test or ANOVA.

Next, the role of Plin1 in LD stabilization and HSC activation was explored. Fresh HSCs were transfected with Ctrl empty or Plin1 expression plasmids 12 h after seeding, and then cultured for 1, 4, or 7 days (Supplementary Fig. 10h). Cellular neutral lipid content analysis showed that Plin1 overexpression increased lipid contents and alleviated their rapid loss (Fig. 8a). Additionally, cells transfected with *Plin1* exhibited decreased levels of various pro-fibrotic genes (Fig. 8b). Staining analysis by Bodipy, Nile red, and αSMA further confirmed the role of *Plin1* overexpression in LD stabilization and HSC activation (Fig. 8c). Moreover, fresh HSCs were transfected with *Plin1* siRNA to further validate its role in LD storage and HSC activation. Fresh HSCs were transfected with respective siRNA 12 h after seeding and then cultured for 1, 4, or 7 days (Supplementary Fig. 10i). As expected, Plin1 deficiency reduced the lipid accumulation and promoted HSC activation (Fig. 8d–f). These results support the view that Plin1 plays a crucial role in maintaining LD stabilization and preventing HSC activation.

**Fig. 9:**
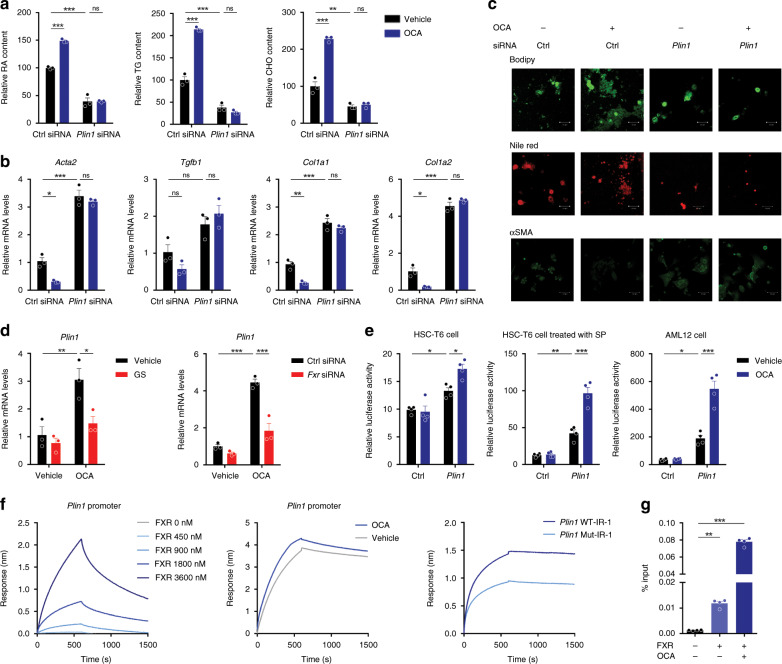
OCA stabilizes LD and inhibits HSC activation via FXR-Plin1 pathway. **a**–**c** The effect of OCA in precluding LD loss and HSC activation depends on Plin1 (*n* = 3 biologically independent samples). Cells transfected with ctrl or *Plin1* siRNA were treated with OCA. **a** Lipids quantitation. **b** mRNA expression of pro-fibrotic genes. **c** Bodipy, Nile red, and α-SMA staining of cells (representative of *n* = 3 biologically independent samples. Scale bar, 50 μm). **d** FXR antagonist and FXR knock-down abolished the upregulation of Plin1 by OCA (*n* = 3 biologically independent samples). **e** Reporter gene analysis of the *Plin1* promoter in HSC-T6 cells in the absence or presence of SP, and in AML-12 cells (*n* = 4 biologically independent samples). **f** BLI analysis for the association between recombinant human FXR protein and *Plin1* promoter. **g** ChIP analysis for the association between endogenous FXR and *Plin1* promoter (*n* = 4 biologically independent samples). Results are mean ± SEM, *P < 0.05, **P < 0.01, ***P < 0.001, and ns, statistically not significant, as assessed with ANOVA.

The possibility that FXR agonists inhibit HSCs activation via regulating Plin1 was examined using freshly isolated HSCs transfected with scramble or *Plin1-*specific siRNA and treated with OCA (Supplementary Fig. 10j). OCA treatment significantly increased the storage of lipids in ctrl siRNA-treated cells, while this effect was abolished in *Plin1* siRNA-treated cells (Fig. 9a, b). Consistently, the effects in decreasing α-SMA level and other pro-fibrotic biomarkers were also abolished in *Plin1* siRNA-treated cells (Fig. 9b, c). Together, these results indicate OCA may inhibit HSC activation via regulating Plin1. OCA is an FXR agonist, and thus the question arises whether *Plin1* is a direct FXR target gene. FXR antagonist and siRNA interference largely abolished the effect of OCA in upregulating *Plin1* (Fig. 9d). Based on previous studies, most functional binding sites (FXRE) identified in FXR target genes correspond to two inverted repeats spaced by one nucleotide as exemplified by the IR-1. The typical IR-1 elements including GGGTGAATAACCT and GGGTCAGTGACCT^48^. Analysis of the proximal promoter of rat *Plin1* gene identified a potential IR-1 (5′-GTGGCAATCACCT-3′) located 1363–1375 bp upstream of the transcription start site. We then cloned this putative *Plin1* gene promoter and evaluated their regulation by FXR. Transactivation of the *Plin1* gene promoter by FXR was evaluated by luciferase reporter gene assays. As expected, the *Plin1* gene promoter was transactivated by FXR in the presence of OCA (Fig. 9e) in HSC-T6 cells. Considering HSCs during the process of promoter transfection have been already activated and thereby compromising the response to FXR agonists, the reporter gene assays were conducted in HSCs in the presence of SP and in AML-12 cells. As expected, much stronger responses to OCA treatment were observed in these conditions (Fig. 9e). BLI assays were conducted to confirm the recruitment of recombinant FXR protein onto the *Plin1* gene promoter. With the increase of FXR protein concentration, the association between FXR protein and the *Plin1* gene promoter was enhanced (Fig. 9f). Moreover, this association was obviously reinforced in the presence of OCA and impaired by the mutation in this putative IR-1 region (Fig. 9f), supporting that FXR protein binds to IR-1 located in *Plin1* promoter. To further validate this association between endogenous FXR and *Plin1* promoter in cells, ChIP assay was further performed to detect whether endogenous FXR binds to *Plin1* promoter. As expected, *Plin1* was successfully identified from the ChIP assay, and the levels of *Plin1* promoter recruited to FXR was significantly elevated following OCA treatment (Fig. 9g). These results support the contention that *Plin1* is a direct FXR target gene.

### FXR SUMOylation represses Plin1 regulation in activated HSCs

**Fig. 10:**
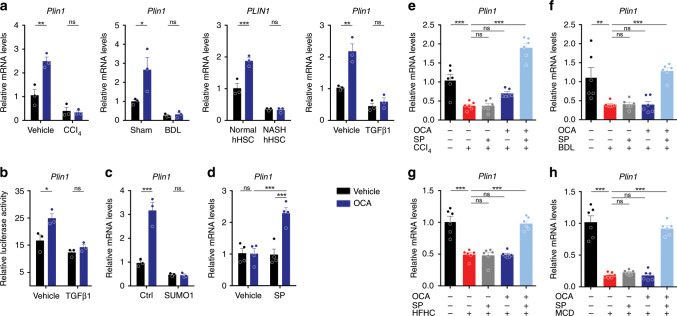
SUMOylation of FXR represses Plin1 transcription. **a** OCA failed to upregulate the *Plin1* mRNA in activated HSCs caused by CCl_4_ treatment, BDL operation and from NASH patients (*n* = 3 biologically independent samples within these experiments). **b** OCA failed to promote the induction of *Plin1* in activated HSCs as reflected by reporter gene assays (*n* = 3 biologically independent samples). **c** SUMOylation of FXR inhibited the transcription of *Plin1* (*n* = 3 biologically independent samples). **d** SP rescued the regulation of OCA on Plin1 in cultured HSCs (*n* = 4 biologically independent samples). **e**–**h** SP treatment restored the regulation of OCA on Plin1 in isolated HSCs from CCl_4_ **e**, BDL **f**, HFHC **g**, or MCD **h** mice (*n* = 6 biologically independent samples within these experiments). Results are mean ± SEM, *P < 0.05, **P < 0.01, and ns, statistically not significant, as assessed with ANOVA.

The present results showed that the increased SUMOylation of FXR in activated HSCs is an important causal factor restricting the functional benefits of FXR agonists against HSC activation and thereby liver fibrosis. It is thus reasonable to predict that the effects of FXR agonists on upregulating *Plin1* may be compromised by increased FXR SUMOylation. In line with the results of the typical FXR target gene *Shp* (Fig. 3a), OCA significantly upregulated *Plin1* in quiescent but not activated HSCs (Fig. 10a). Similar results were observed in reporter gene assays (Fig. 10b). Additionally, OCA treatment failed to upregulate *Plin1* in cells transfected with the SUMO1 expression vector plasmid (Fig. 10c). In contrast, the SUMOylation inhibitor SP significantly restored the upregulation of *Plin1* by OCA in activated HSCs (Fig. 10d). Moreover, in freshly isolated HSCs from CCl_4_-, BDL-, or NASH-induced fibrotic mice, therapeutic administration of OCA could up-regulate *Plin1* expression in the presence of SP (Fig. 10e–h). Collectively, these results suggest that the increased FXR SUMOylation may compromise the functional benefits of FXR agonists in activating *Plin1* and thereafter the efficacies in inhibiting HSC activation and fibrotic development.

## Discussion

FXR is a promising target in clinical trials for the treatment of liver fibrosis. In phase III clinical trials, OCA, a potent FXR agonist, has reached one of the primary endpoints in NASH-associated fibrosis. However, the response rate of OCA treatment to NASH-induced liver fibrosis is only 23%. Therefore, it is necessary to exploit new approaches to improve the clinical benefits of OCA and other FXR agonists. Here, we find that enhanced SUMOylation of FXR in activated HSCs of fibrotic livers is a key factor restricting the functional benefits of OCA and other FXR agonists and validate that SUMOylation inhibitors combined with OCA potentiates the efficacy of OCA in treatment of liver fibrosis in CCl_4_, BDL, and NASH models. Mechanistically, we find that Plin1, a key protein in stabilizing LDs, is a direct target gene of FXR in HSCs. FXR agonists elicit their clinical benefits to retarding liver fibrosis mainly via upregulating Plin1 and thereby stabilizing LDs for preventing HSCs activation.

FXR agonists have shown anti-fibrotic/cirrhotic effects in several rodent models, including CCl_4_ and TAA poison, BDL, porcine serum injection, and NASH^38,49^. Recently, FXR agonists were also found to improve portal hypertension in cirrhotic rats^35,50^. These pre-clinical studies support the notion that FXR agonists are promising for therapy of liver fibrosis. However, results from several clinical trials in PBC patients showed only marginal anti-fibrotic activity for OCA^21,22^. Although OCA was validated in several clinical trials for its benefit in ameliorating NASH-induced liver fibrosis^51,52^, the response rate was 23%, far from satisfactory^19,20^. The causes underlying the poor translation of preclinical results from animal models to clinical patients can be very complex. Notably, therapeutic agents have been administered before the real development of liver fibrosis in most of the preclinical researches, which is different from that in clinical patients. We recently identified that the protein level of FXR in the process of fibrosis is gradually reduced which may partially explain why the functional benefits of FXR agonists to fibrosis of clinical patients are limited^23^. In this study, we validated that prophylactic but not therapeutic administration of OCA exerted sufficient anti-fibrotic effects in CCl_4_-, BDL- and NASH-induced liver fibrosis, suggesting that the functional response of FXR to its agonists may be compromised during the process of fibrogenesis.

The fibrotic development of liver can be attributed to multifaceted factors because of differential pathological causes to liver damage; however, it has been widely acknowledged that activation of HSCs represents a hallmark of fibrosis independent of the etiology in liver damage^53,54^. FXR agonists may impede fibrogenesis via multiple mechanisms as previously described. However, previous reports about whether and how FXR regulates the activation of HSCs were inconsistent. Some studies demonstrated that FXR was positively expressed and functionally active in HSCs^37^. However, several other studies claimed that HSCs showed limited functional response upon FXR agonist exposure^35^. The current study found that exposure of OCA to quiescent HSCs but not activated cells significantly increased FXR signaling. Moreover, OCA pretreatment is effective against HSCs activation but showed little effect on pre-activated HSCs. These results may explain previous discrepancies in the literature and support our view that the response of FXR to its agonists depends on the stage of disease. In activated HSCs and in the process of liver fibrogenesis, the functional response of FXR is gradually reduced compromising the functional benefits of FXR agonists.

Our recent study indicated that FXR protein in the liver is gradually lost in the process of liver fibrogenesis^23^. However, we found that the FXR protein level in HSCs remained largely unchanged. We thus surveyed the PTMs of FXR and found that SUMOylation was increased in activated HSCs and fibrotic livers. In addition, increased SUMOylation was also found in primary HSCs from NASH mice and patients, and we found that SUMOylation of FXR in HSCs was mainly mediated by SUMO1 at Lys^122^, Lys^275^, and Glu^277^. Moreover, enhanced FXR SUMOylation accounts for the loss of the functional response of HSCs to FXR agonists. In line with the current results, others demonstrated that SUMOylation of FXR negatively regulated expression of its target genes, including *SHP*, *BSEP*, and *OSTB*^55^. Combination of SUMOylation inhibitors with OCA treatment resulted in inhibition of HSC activation and fibrogenesis in both CCl_4_, BDL, and NASH models. It is thus optimistic to expect that SUMOylation inhibitors like SP and GA together with FXR agonists can be a therapeutic approach to treat liver fibrosis of various etiologies. SP is a natural product aminocyclitol produced by *Streptomyces spectabilis*. It is a potent inhibitor of bacterial ribosomes and is accepted as therapeutic for respiratory tract and sexually transmitted infections^56^ with an excellent safety profile^57^. GA, also known as anacardic acids, is a botanical drug isolated from the seed coat of *Ginkgo biloba* L. with a wide range of bioactive properties, including anti-bacterium, anti-HIV, and molluscicidal activities. Both SP and GA exert excellent role in inhibiting protein SUMOylation^58,59^, and are therefore readily translated to the clinical use.

During the development of liver fibrosis, the levels of various BA species generated in hepatocytes are dramatically increased, which could promote the activation and proliferation of HSCs. Activation of FXR in hepatocytes inhibits the biosynthesis of BAs, contributing in part to its anti-fibrotic activity^39^. However, as results from the OCA clinical trial in PBC patients indicated, OCA was not effective against PBC-associated fibrosis^21,22^, suggesting that reducing BA accumulation may not be major mechanism underlying the functional benefits of FXR for liver fibrosis. HSCs are key contributors in the development of liver fibrosis. Activation of FXR in HSCs results in up-regulation of SHP, which binds JunD and inhibits DNA binding of adaptor protein-1^37^. Activation of FXR in HSCs results in up-regulation of PPARγ, and co-treatment with OCA potentiates the anti-fibrotic activity of rosiglitazone, a PPARγ ligand^36^. Therefore, FXR-SHP and the FXR-PPARγ cascade may contribute to inhibit HSC activation. During HSC activation, cytoplasmic LDs are degraded, which is believed to be functionally linked to expression of pro-fibrogenic genes and its subsequent fibrogenesis. Thus, modulating the lipid metabolism and maintaining LD structure were proposed as a strategy to prevent HSCs activation and impede liver fibrosis. Patatin-like phospholipase domain-containing 3 (PNPLA3), a lipid-metabolizing enzyme, is required for HSC activation, and the genetic variant of PNPLA3 I148M conferred proinflammatory and profibrogenic properties to HSCs^60^. Liver fatty acid-binding protein (FABP1), a cytosolic protein involved in the uptake, transport, and metabolism of fatty acids, regulates the fibrogenic program of HSCs^61^. Rab18, a RA-responsive LD-associated protein, helps mediate HSCs activation^45^. Plin1, one of the major LD-binding proteins, is highly expressed in lipid-enriched cells, such as adipocytes^62^. In quiescent adipocytes, Plin1 provides a protective barrier against lipase activities and LD loss^63^. This gatekeeper role for Plin1 is also found in HSCs in our study. Plin1, together with other perilipin family members, are abundantly expressed in freshly isolated HSCs, with a coordinated decrease in *Plin1* mRNA expression during the culture of these cells, and that these changes are closely related to LD depletion and HSC activation. On the contrary, overexpression of Plin1 promotes accumulation of LD and inhibits HSCs activation. These findings uncovered Plin1 as a therapeutic target in preventing HSC activation and fibrosis development. More importantly, Plin1, but not other perilipin members, is transcriptionally regulated by FXR in HSCs. Exposure of FXR agonist to quiescent HSCs significantly promotes *Plin1* transcription and prevents HSCs activation which is dependent on Plin1. However, SUMOylation of FXR in activated HSCs repressed its regulation of *Plin1*, resulting in diminished benefits of FXR agonists against HSC activation and fibrosis development.

In this study, we demonstrate that FXR agonists prevented HSC activation and LD loss via regulation of Plin1, indicating that the FXR-Plin1 cascade can be an important target for drug discovery and therapeutics exploitation. Notably, enhanced SUMOylation of FXR in the process of HSCs activation and fibrogenesis strongly compromises FXR signaling, providing insights into understanding why OCA alone has limited effects against liver fibrosis. More importantly, the present study provides a promising strategy of combined use of FXR agonists with SUMOylation inhibitors for the therapy of liver fibrosis of various etiologies including toxins, cholestasis, and particularly NASH.

## Methods

### Reagents

OCA, GW4064, WAY-362450, ginkgolic acid (GA), and spectinomycin (SP) were purchased from MedChem Express (NJ, USA). Carbon tetrachloride (CCl_4_) and mineral oil were from Sigma-Aldrich (St. Louis, MO, USA). Z-guggulsterone (GS) was obtained from APExBIO (Houston, TX, USA).

### Animals and treatment

Male C57BL/6J mice (8 weeks old, 20 g) were obtained from Vital River Laboratory Animal Technology Co. Ltd. (Beijing, China). The animal studies were approved by the Animal Ethics Committee of China Pharmaceutical University. All mice were kept in an air-conditioned animal quarter at a temperature of 25 ± 2 °C and a relative humidity of 50 ± 10% with 12-h light/dark cycles for 1 week before experiments, and allowed water and standard chow ad libitum.

To determine whether prophylactic or therapeutic administration of OCA was able to rescue liver fibrosis, mice received intraperitoneal (i.p.) injection of CCl_4_ (20% CCl_4_/mineral oil; 5 ml/kg) or mineral oil three times per week for 4 weeks in total^23^. In the prophylactic arms, OCA (1.5 or 5 mg/kg) was administered every other day for 4 weeks; while in the therapeutic arms, OCA was administered at same dose daily from the 3rd week for 2 weeks. Mice in these two arms received equal amounts of OCA. This effect of OCA was further validated in the bile duct ligation (BDL)-induced fibrosis model. BDL was conducted as previously described^6^. Briefly, mice were anesthetized and subjected to double ligation of the common bile duct below the bifurcation, while sham-treated mice underwent the same procedure with bile duct exposure, but without ligation. In the prophylactic arms, OCA (1.5 or 5 mg/kg) was administered for 14 days every other day after BDL operation; while in therapeutic arms, same dose of OCA was administered daily from the 7th day after BDL operation. Mice in these two arms received equal amount of OCA, which was suspended in 1% sodium methyl cellulose for gavage administration. This discrepancy in the anti-fibrotic effect of OCA were further validated in NASH-induced fibrosis models. Mice were fed with high fat (40%) plus high CHO (0.2%) diet (Trophic Animal Feed High-tech Co., Ltd, Jiangsu, China) and fructose/sucrose (23.1 and 18.9 g/L, respectively) in drinking water (HFHC) for 16 weeks to induce NASH and fibrosis. In the prophylactic arms, OCA (1.5 or 5 mg/kg) was administrated from the 9th week for 8 weeks every other day; while in therapeutic arms, same dose of OCA was administrated from 13th weeks for consecutive 4 weeks daily. Another NASH and fibrosis model caused by MCD diet (Trophic Animal Feed High-tech Co., Ltd) feeding was also included in this study. In the prophylactic arms, OCA (1.5 or 5 mg/kg) was administrated from the first week to the 6th week every other day; while in therapeutic arms, same dose of OCA was administrated from the 4th week to the 6th week every day. Mice in these two arms received equal amount of OCA. These experiment procedure schemes are present in corresponding figures.

To determine the effect of co-administration of OCA and SP on liver fibrosis, the mice were treated with CCl_4_ or BDL as described above. SP, dissolved in phosphate buffered saline, was subcutaneously injected at dosage of 200 mg/kg/day. OCA was administered 1.5 or 5 mg/kg from the 3rd week after CCl_4_ treatment or from the 7th day following BDL. Mice were fed with HFHC diet and fructose/sucrose drinking water for 16 weeks. From the 13th week, these mice were treated with OCA with or without SP until the end of the procedure. Otherwise, mice were fed with MCD diet for 6 weeks and treated with OCA with or without SP from the 4th week. These experimental procedure schemes are present in corresponding figures.

### Serum biochemical analysis

Serum levels of alanine aminotransferase (ALT) and aspartate aminotransferase (AST) were determined by an automatic blood biochemical analyzer (Beckman Counter LX20, USA).

### Histological analysis

Formalin-fixed liver tissues were embedded in paraffin and 5 μm-thick sections were cut for H&E, Masson’s trichrome, and Sirius red staining.

### Cell isolation, culture, and treatment

HSC-T6 cells were obtained from Central South University (Changsha, China) and AML12 cell line was purchased from Cell Bank of Shanghai Chinese Academy of Sciences (Shanghai, China). Primary human HSCs from healthy donors or NASH-induced fibrotic patients were purchased from ZenBio (Research Triangle Park, NC, USA) and iXCells Biotech (San Diego, CA, USA), respectively. Primary human HSCs between passages 2 and 5 were used. Primary murine HSCs were isolated by pronase–collagenase perfusion and density gradient centrifugation as previously described^23^. Briefly speaking, primary HSCs were isolated by pronase/collagenase digestion followed by single-step density gradient centrifugation with Nocodazole. Cells were cultured in Dulbecco modified Eagle’s medium (DMEM) containing 10% fetal calf serum, 100 U/ml penicillin, and 100 μg/ml streptomycin (Invitrogen, Thermo Fisher, MA, USA) in a humidified atmosphere of 5% CO_2_ at 37 °C. The purity of the HSC fraction (~95%) was assessed by auto-fluorescence. For SUMO inhibition studies, primary HSCs were treated with vehicle or a SUMO inhibitor (GA 50 μM or SP 5 μM) from day 1 and then treated with FXR agonists (OCA, GW4064 or WAY-362450 at 5 μM) at day 4. In GS studies, cells were treated with GS (20 μM) for 0.5 h before receiving either vehicle or OCA. For siRNA transfection studies, the cells were transfected with *Fxr*-specific siRNA, *Plin1*-specific siRNA, or scramble siRNA using lipofectamine RNAiMAX reagent (Invitrogen) for 48 h before OCA treatment, according to the manufacturer’s protocols. According to previous reports, K122R, K275R, or E277A FXR single mutants or K122R/K275R/E277A FXR triple mutant plasmids were produced, respectively^43^. For plasmid transfection studies, the cells were transfected with control, Plin1, SUMO1, FXR WT, or mutants using Lipofectamine 2000 reagent (Invitrogen) according to the manufacturer’s protocols.

### Cellular lipid staining and quantification

HSCs were stained with Nile Red reagents (MCE) and Bodipy493/503 (Thermo Fisher) to visualize the lipids with a light microscope. Cellular RA quantitative detection was managed by RA ELISA kit (Shanghai Yuanmu Biological Technology Co. Ltd., Shanghai, China) according to the manufacturer’s protocol. Cellular TG and CHO levels were detected by commercially available kits (Nanjing Jiancheng Bioengineering Institute, Jiangsu, China) following the manufacturer’s protocols.

### RT-PCR

Total RNA was prepared using RNAiso Plus reagent (TaKaRa Biotechnology, Dalian, China) and analyzed by real-time polymerase chain reaction (RT-PCR) as previously described^64^. The primers are list in Supplementary Table 1.

### Western blot analysis

Western blot analysis was conducted with standard method as previously described^65^. Briefly, protein lysates were separated by SDS–PAGE and transferred to a PVDF membrane, which was then blocked in 5% nonfat milk. The blots were incubated with primary antibodies and appropriate secondary antibodies, and detected by enhanced chemiluminescence kit (Thermo Fisher Scientific, Waltham, MA, USA). Primary antibody against FXR (AB10304) was purchased from Millipore (Billerica, MA, USA). Primary antibodies against Plin1 (ab61682) and α smooth muscle actin (αSMA, ab5694) were purchased from Abcam (Cambridge, UK, USA). Primary antibodies against Phospho-Tyrosine (9411), Acetylated-Lysine (9441), and SUMO1 (4930S) Cell Signaling Technology (CST, Danvers, MA, USA). Primary antibody against SUMO2 (BML-PW9465-0100) and GAPDH (AP0063) were purchased from Enzo Life Sciences (Switzerland) and Bioworld Technology (Bloomington, USA), respectively. The uncropped and unprocessed scans blots are present in the Source Data file.

### Co-immunoprecipitation (Co-IP)

Co-IP analysis was conducted with standard method as previously described^23^. The immunoprecipitates were collected for SDS–PAGE separation and immunoblotting.

### In vivo SUMOylation assays

In vivo SUMOylation assays were performed using the EpiQuik In Vivo Protein Sumoylation Assay Ultra Kit (Epigentek, Farmingdale, NY, USA). In brief, nuclear extracts were prepared using the Nuclear and Cytoplasmic Extraction Kit (Beyotime, Jiangsu, China) according to the manufacturer’s protocol. Extracts were immunoprecipitated with anti-SUMO1 antibody followed by anti-FXR antibody.

### Biolayer interferometry (BLI) assay

The association of recombinant human FXR protein to the promoter of Plin1 was confirmed by BLI using an OctetRED 96 instrument (ForteBio). The wild type (WT) and mutant oligonucleotides used in this studies were as follows: Plin1 WT-IR-1, 5′- GAACTCAAGCGGTCAGGCTTG**GTGGCAATCACCT**TGACCTGCTGAGCCATTTTGG-3’ (the IR-1 is indicated in *boldface* type), Plin1 Mut-IR-1, 5′- GAACTCAAGCGGTCAGGCTTG**GTGGCAATCAaaT**TGACCTGCTGAGCCATTTTGG-3’ (the IR-1 is indicated in *boldface* and the mutant is in lowercase type). The biotinylated-DNA containing Plin1 promoter was loaded onto streptavidin optical biosensors and incubated with FXR protein in the presence or absence of OCA. The results were processed and the association and dissociation plot and kinetic constants (*K*_on_ and *K*_off_) were obtained from ForteBio data analysis software. Equilibrium dissociation constants (*K*_D_) were calculated by the ratio of *K*_off_ to *K*_on_^23^.

### Reporter gene analysis

Cells were transfected with Plin1 luciferase reporter constructs using Lipofectamine 2000 reagent according to the manual instruction and treated with OCA in the absence or presence of SP for 24 h. Cells were lysed and the luciferase activities were measured with the Luc-Pair Duo-Luciferase HS Assay Kit (GeneCopoeia, Rockville, MD, USA)^65^.

### Chromatin immunoprecipitation (ChIP) assay

The HSC-T6 cells were prepared for the ChIP assay using a SimpleChIP Plus Sonication Chromatin IP kit (CST) according to the manufacturer’s protocols. Immunoprecipitation was performed with anti-FXR antibody (sc-25309×, Santa Cruz Biotechnology, Dallas, TX, USA) and normal IgG (401501, BioLegend, San Diego, CA, USA). The resulting precipitated DNA specimens were analyzed using the following primers for *Plin1* promoter: forward, 5′-TTTGGAAGCTCCTTGCTC-3′ and reverse, 5′-CAGATAGATCCTTGGTGG-3′.

### Statistical analysis

Data were analyzed using GraphPad Prism (Graphpad Software, Inc., San Diego, CA, USA) and are presented as the mean ± standard error of mean (SEM). A two-tailed Student’s *t*-test was applied for comparison of two groups and a one-way ANOVA with Tukey post hoc analysis was applied for comparison of multiple groups. *P* values below 0.05 were considered statistically significant.

### Reporting summary

Further information on research design is available in the Nature Research Reporting Summary linked to this article.

## Supplementary information

Supplementary Information

Reporting Summary

## Source data

Source Data

## Data Availability

Source data for Figs. 1–10 and Supplementary Figs. 1–10 are provided with the paper. All data are available from the corresponding author upon reasonable request.
