# Supplementary material for: SUMOylation inhibitors synergize with FXR agonists in combating liver fibrosis
Source: Nat Commun. 2020 Jan 13;11:240. doi: 10.1038/s41467-019-14138-6 (PMC6957516; doi:10.1038/s41467-019-14138-6)
Supplement: Supplementary file 1 — Supplementary Information [file 41467_2019_14138_MOESM1_ESM.pdf]

Supplementary Information

SUMOylation inhibitors synergize with FXR agonists in combating liver fibrosis

Zhou et al.

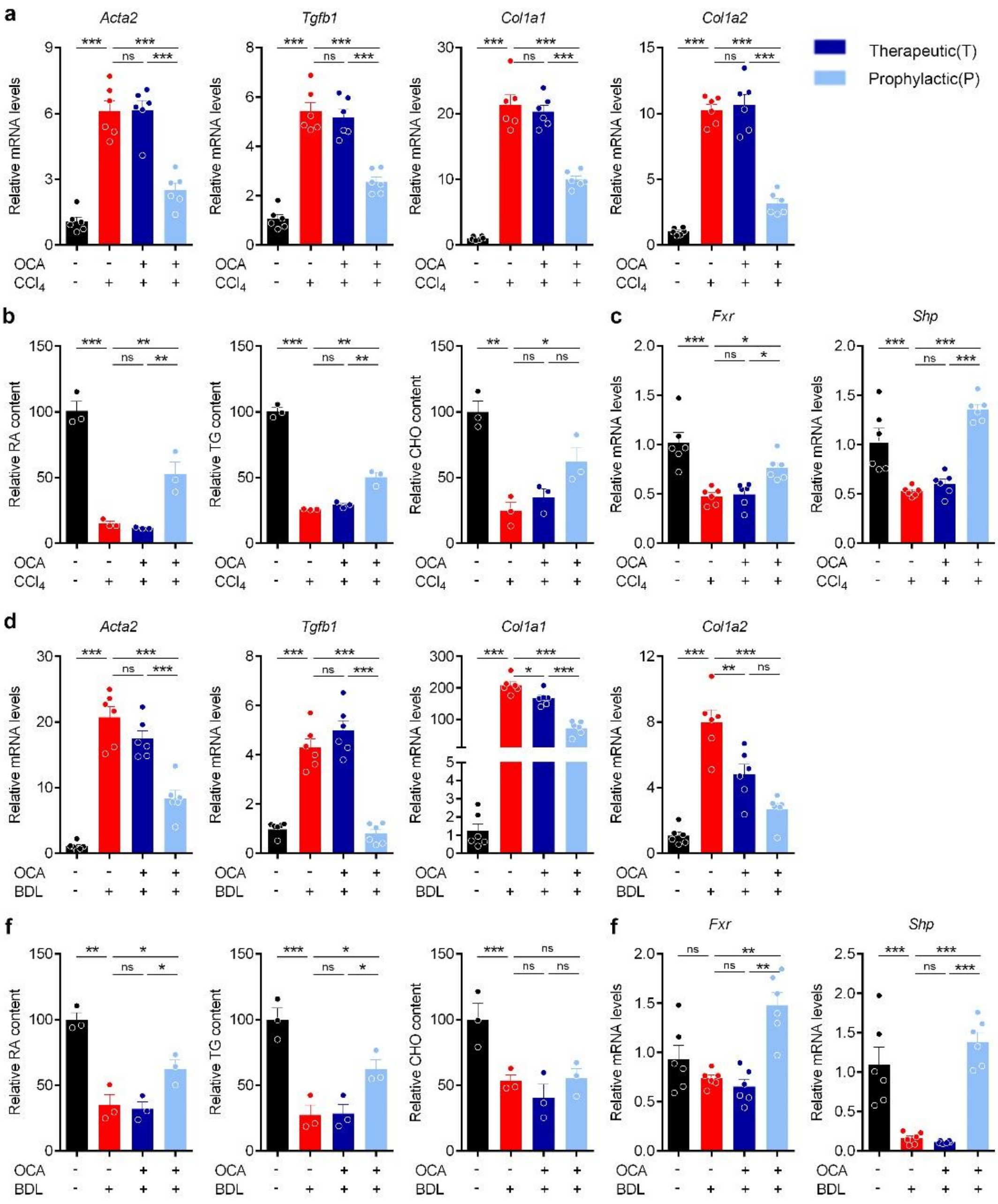

Supplementary Fig. 1. **Prophylactic but not therapeutic administration of OCA inhibits HSC activation and LD loss**. Anti-fibrotic effects of prophylactic and therapeutic administration of OCA were evaluated in freshly isolated HSCs from CCl_4-induced (**a-c**) or BDL-induced (**d-f**) liver fibrosis models. **a** and **d**, Levels of *Acta2*, *Tgfb1*, *Col1a1* and *Col1a2* mRNAs in freshly isolated HSCs (n=6 biologically independent samples within these experiments). **b** and **e**, lipid quantitation analysis of isolated HSCs (n=3 biologically independent samples within these experiments). **c** and **f**, levels of *Fxr* and *Shp* mRNAs in freshly isolated HSCs (n=6 biologically independent samples within these experiments). Results are mean ± SEM, **P*< 0.05, ***P*< 0.01, ****P*< 0.001, and ns, statistically not significant, as assessed with ANOVA.

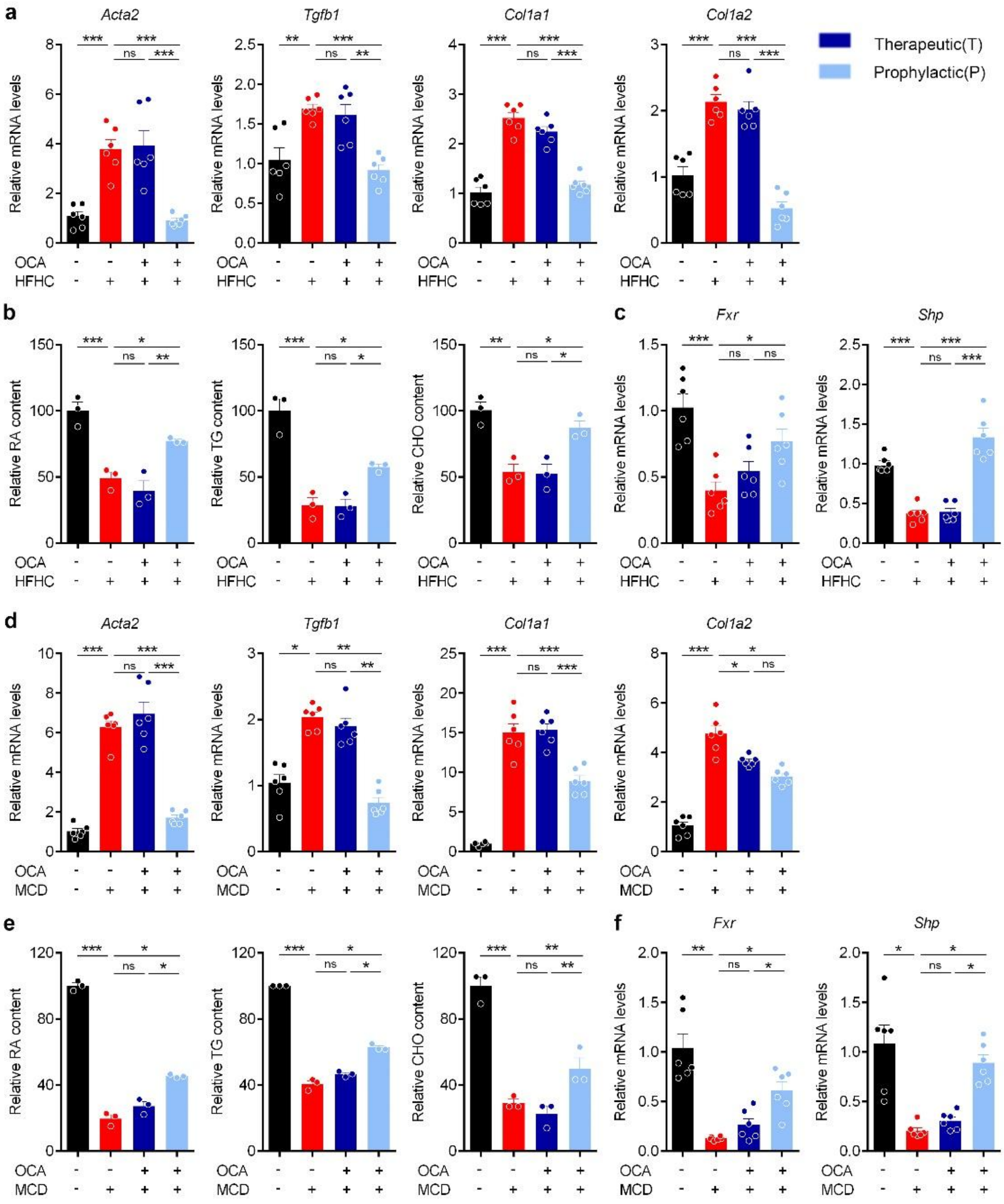

Supplementary Fig. 2. **Prophylactic but not therapeutic administration of OCA inhibits HSC activation and LD loss in NASH models**. Anti-fibrotic effects of prophylactic and therapeutic administration of OCA were evaluated in freshly isolated HSCs from mice fed with HFHC (**a-c**) or MCD diet (**d-f**). **a** and **d**, Levels of *Acta2*, *Tgfb1*, *Col1a1* and *Col1a2* mRNAs in freshly isolated HSCs (n=6 biologically independent samples within these experiments). **b** and **e**, lipid quantitation analysis of isolated HSCs (n=3 biologically independent samples within these experiments). **c** and **f**, levels of *Fxr* and *Shp* mRNAs in freshly isolated HSCs (n=6 biologically independent samples within these experiments). Results are mean ± SEM, *P< 0.05, **P< 0.01, ***P< 0.001, and ns, statistically not significant, as assessed with ANOVA.

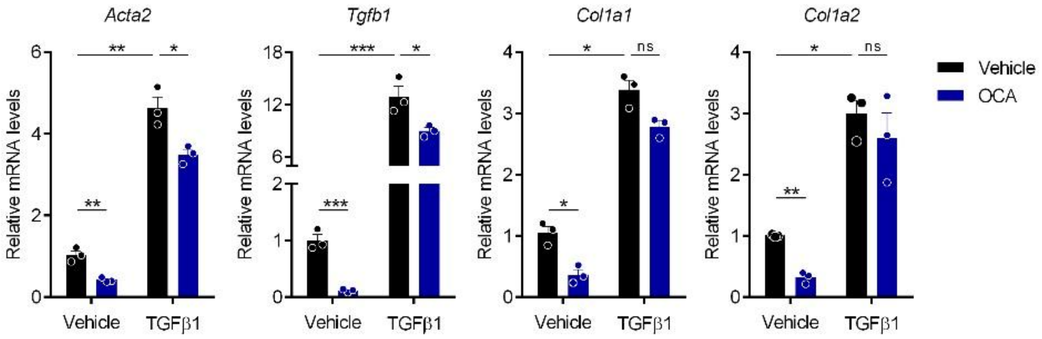

Supplementary Fig. 3. **OCA is ineffective against already activated HSC-T6 cells**. Cultured HSC-T6 cells, exposed to vehicle or TGFβ1, were then treated with OCA. Levels *Acta2*, *Col1a1*, *Col1a2* and *Tgfb1* mRNAs. n=3 biologically independent samples. Results are mean ± SEM, *P< 0.05, **P< 0.01, ***P< 0.001, and ns, statistically not significant, as assessed with ANOVA.

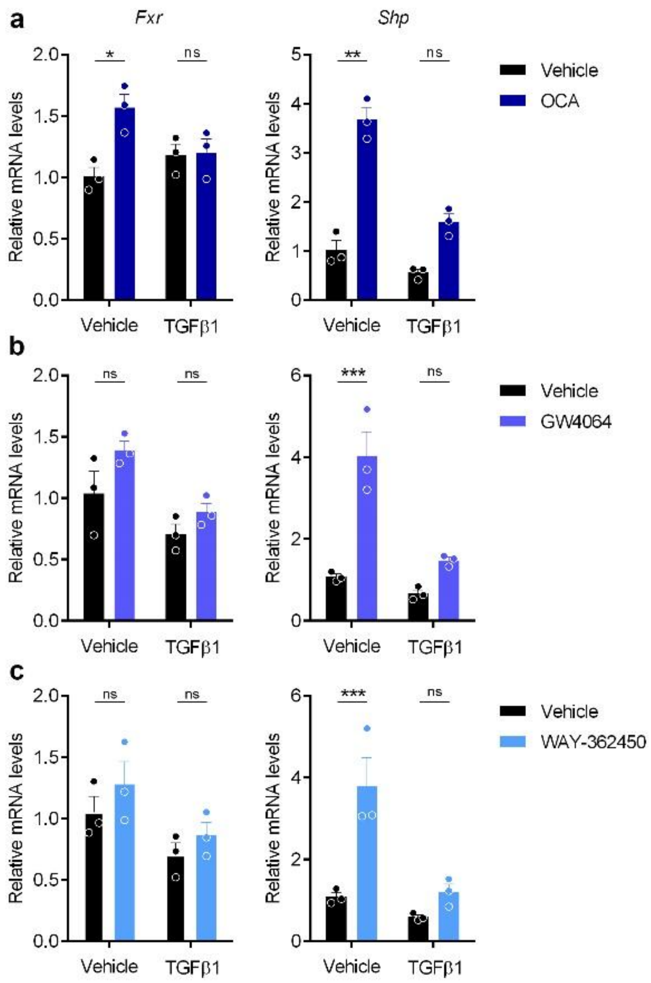

Supplementary Fig. 4. **Activated HSCs show limited functional response to FXR ligands**. Cultured HSC-T6 cells, exposed to vehicle or TGFβ1, were then treated with FXR ligands, including OCA (**a**), GW4064 (**b**), and WAY-362450 (**c**). Levels of *Fxr* and *Shp* mRNAs. n=3 biologically independent samples within these experiments. Results are mean ± SEM, $^{**}P < 0.01$, $^{***}P < 0.001$, and ns, statistically not significant, as assessed with ANOVA.

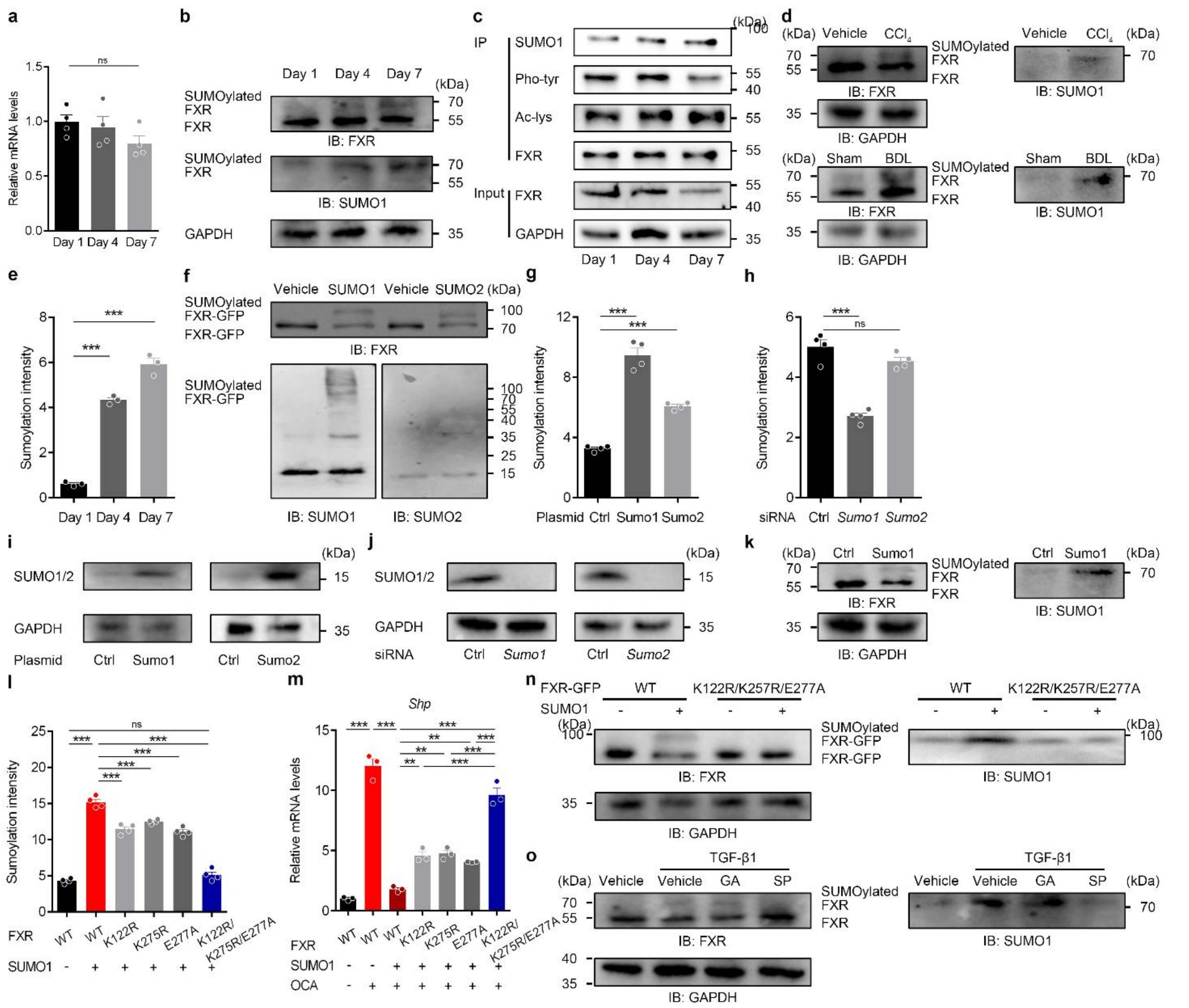

Supplementary Fig. 5. **Expression and PTMs of FXR during the activation of HSCs**. **a**,**b**, mRNA (n=4 biologically independent samples) (**a**) and protein (**b**) levels of FXR in primary HSCs cultured for 1, 4, and 7 days. **c**, PTMs of FXR in primary HSCs cultured for 1, 4, and 7 days as analyzed by Co-IP. **d**, **e**, SUMOylation of FXR in primary HSCs cultured for 1, 4, and 7 days as analyzed by western blot (**d**) and Protein SUMOylation Assay Ultra Kit (**e**) (n=3 biologically independent samples). **f**, SUMOylation analysis of recombinant FXR-GFP protein. **g**, **h**, SUMOylation analysis of FXR in HSCs transfected with Sumo1/2 plasmids (**g**) or siRNA (**h**). **i**, **j**, Protein expression of SUMO1/2 in HSCs transfected with Sumo1/2 plasmids (**i**) or siRNA (**j**). **k**, SUMOylation of FXR in HSCs transfected with Sumo1 plasmid. **l**, **m**, FXR SUMOylation (**l**) and Shp levels (**m**) in cells transfected with WT or SUMO-site mutant FXR plasmids. **n**, FXR SUMOylation by western blot analysis in cells transfected with WT or triple mutant FXR plasmid. **o**, FXR SUMOylation by western blot analysis in cells treated with GA or SP in the presence of TGF-β1. Results are mean ± SEM, **P*< 0.05, ***P*< 0.01, ****P*< 0.001, and ns, statistically not significant, as assessed with ANOVA.

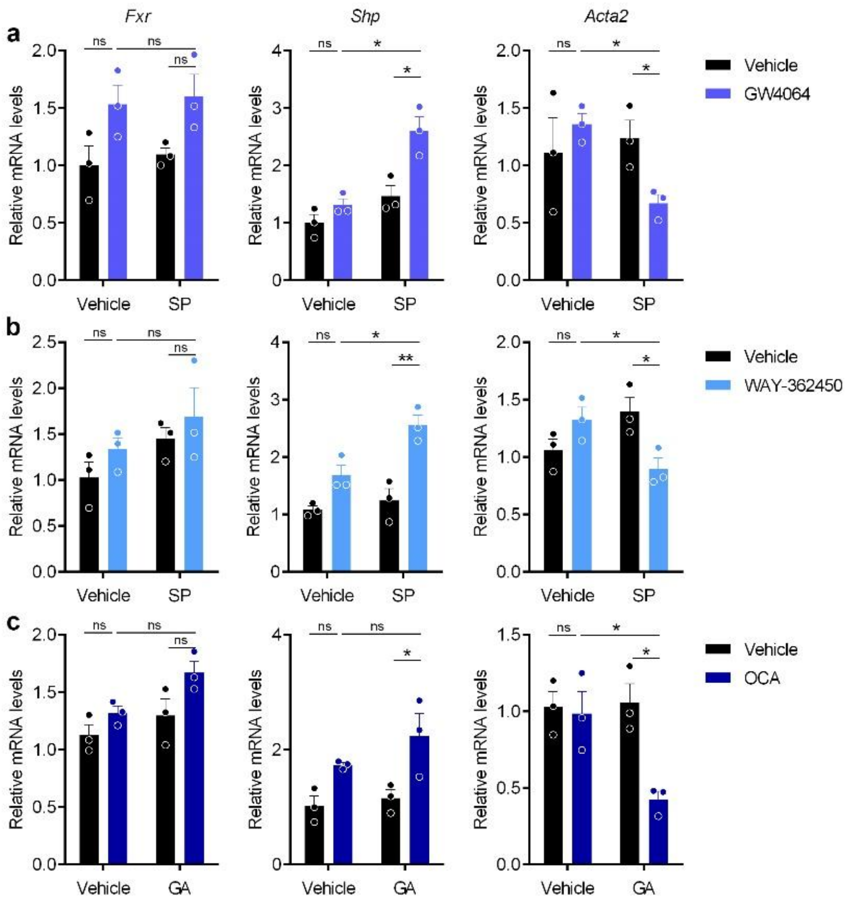

Supplementary Fig. 6. **SUMOylation inhibition restores the activity of FXR in activated HSCs**. Primary HSCs were isolated and cultured for 4 days. These cells, exposed to vehicle or SUMOylation inhibitors, including SP (**a**, **b**) and GA (**c**), were then treated with various FXR ligands, including GW4064 (**a**), WAY-362450 (**b**), and OCA (**c**). Levels of *Fxr*, *Shp* and *Acta2* mRNAs. n=3 biologically independent samples within these experiments. Results are mean ± SEM, **P*< 0.05, and ns, statistically not significant, as assessed with ANOVA.

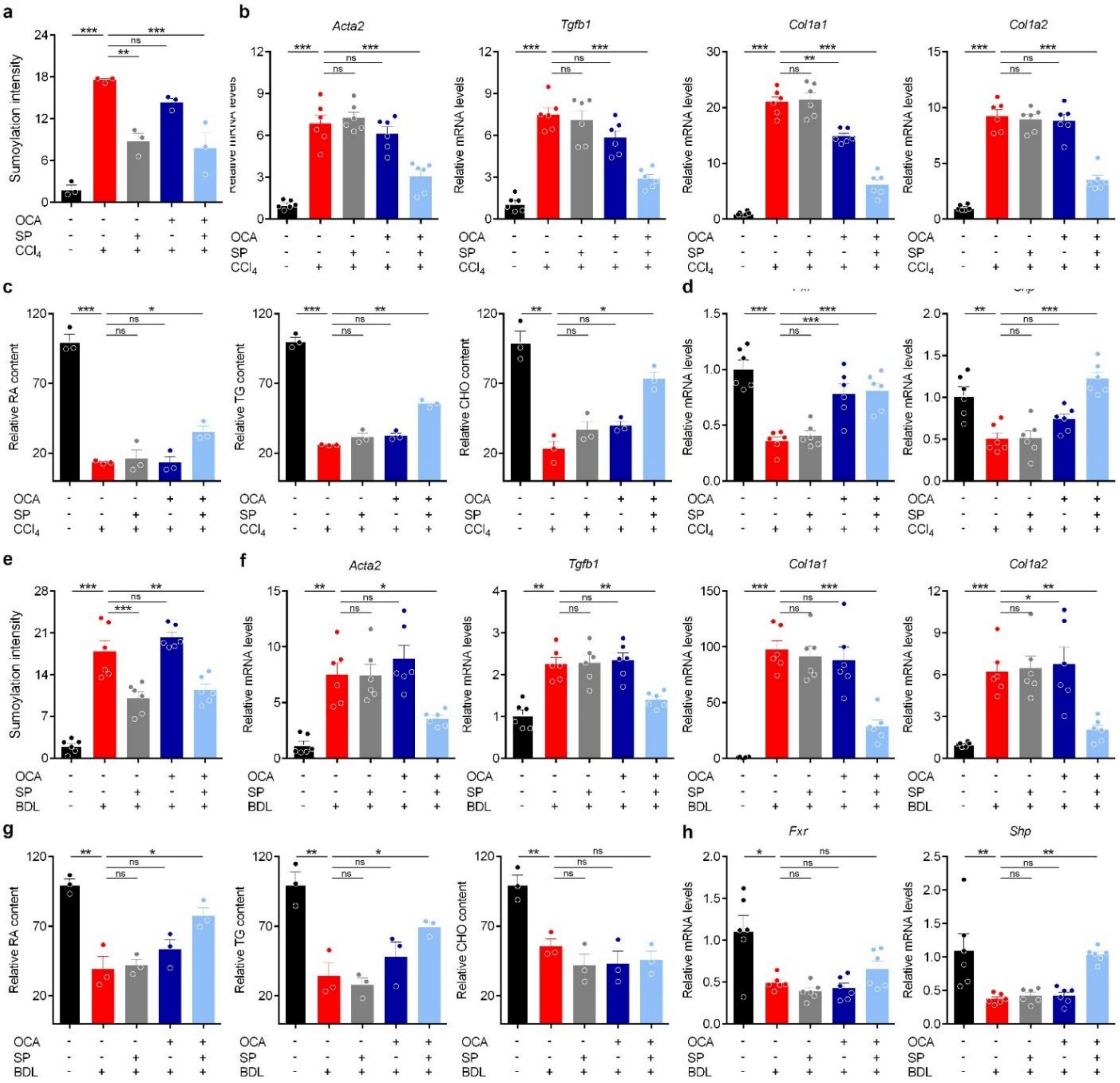

Supplementary Fig. 7. SUMOylation inhibition rescues the effects of FXR agonists in inhibiting HSC activation and LD loss caused by toxin or cholestasis. Fibrotic mice, caused by CCl_4 treatment (**a-d**) or BDL operation (**e-h**), were treated with OCA in the presence or absence of SP. HSCs were then isolated and analyzed. **a** and **d**, SUMOylation of FXR as analyzed by Protein SUMOylation Assay Ultra Kit. **b** and **e**, Levels of *Acta2*, *Col1a1*, *Col1a2* and *Tgfb1* mRNAs in freshly isolated HSCs (n=6 biologically independent samples within these experiments). **c** and **f**, lipid quantitation analysis of isolated HSCs (n=3 biologically independent samples within these experiments). **d** and **h**, Levels of *Fxr* and *Shp* of mRNAs in freshly isolated HSCs (n=6 biologically independent samples within these experiments). Results are mean ± SEM, *$P < 0.05$, **$P < 0.01$, ***$P < 0.001$, and ns, statistically not significant, as assessed with ANOVA.

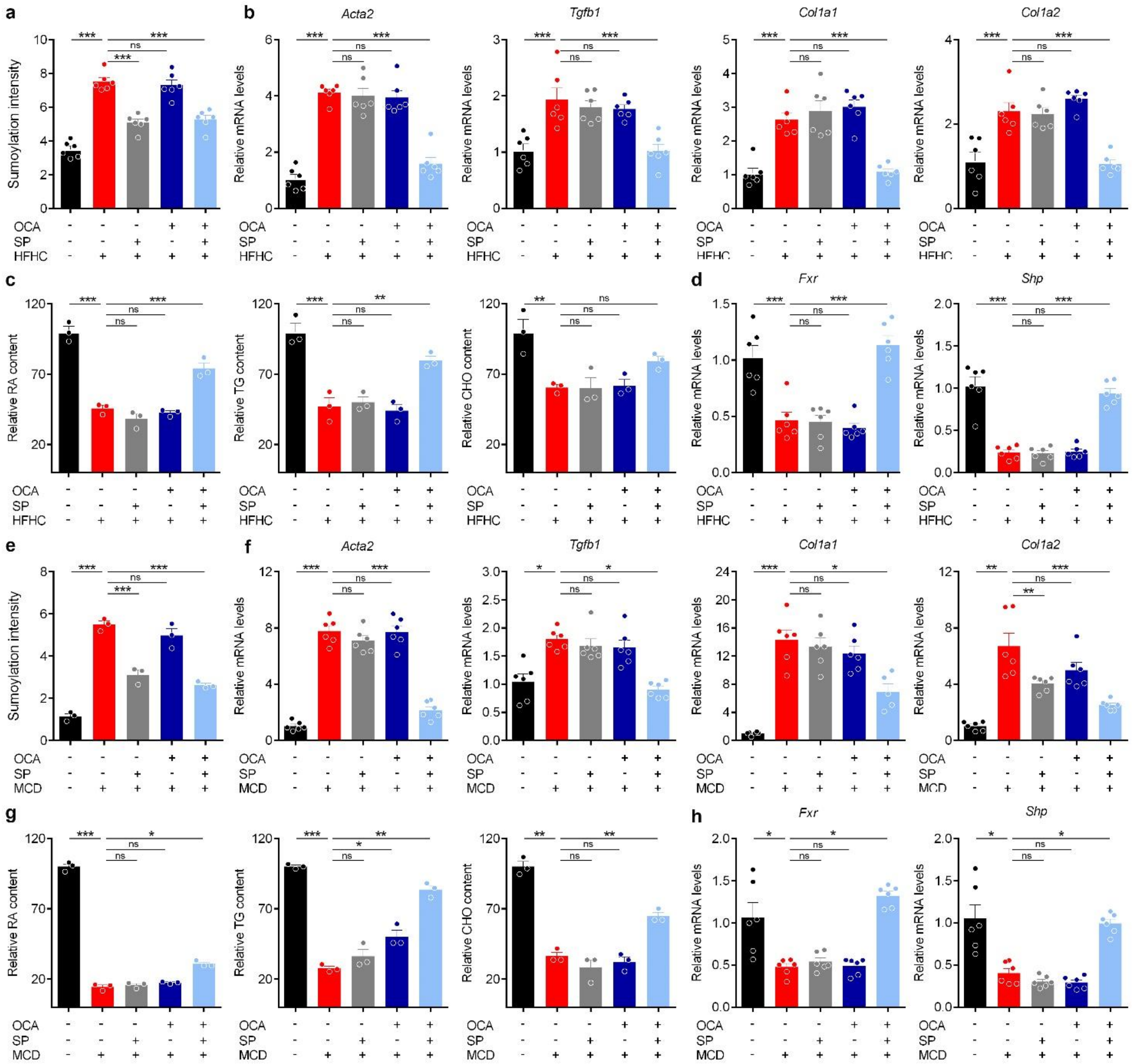

Supplementary Fig. 8. **SUMOylation inhibition rescues the effects of FXR agonists in inhibiting HSC activation and LD loss caused by NASH**. Fibrotic mice, caused by HFHC (**a-d**) or MCD diet (**e-h**), were treated with OCA in the presence or absence of SP. HSCs were then isolated and analyzed. **a** and **d**, SUMOylation of FXR as analyzed by Protein SUMOylation Assay Ultra Kit. **b** and **e**, Levels of *Acta2*, *Col1a1*, *Col1a2* and *Tgfb1* mRNAs in freshly isolated HSCs (n=6 biologically independent samples within these experiments). **c** and **f**, lipid quantitation analysis of isolated HSCs (n=3 biologically independent samples within these experiments). **d** and **h**, Levels of *Fxr* and *Shp* of mRNAs in freshly isolated HSCs (n=6 biologically independent samples within these experiments). Results are mean ± SEM, **P*< 0.05, ***P*< 0.01, ****P*< 0.001, and ns, statistically not significant, as assessed with ANOVA.

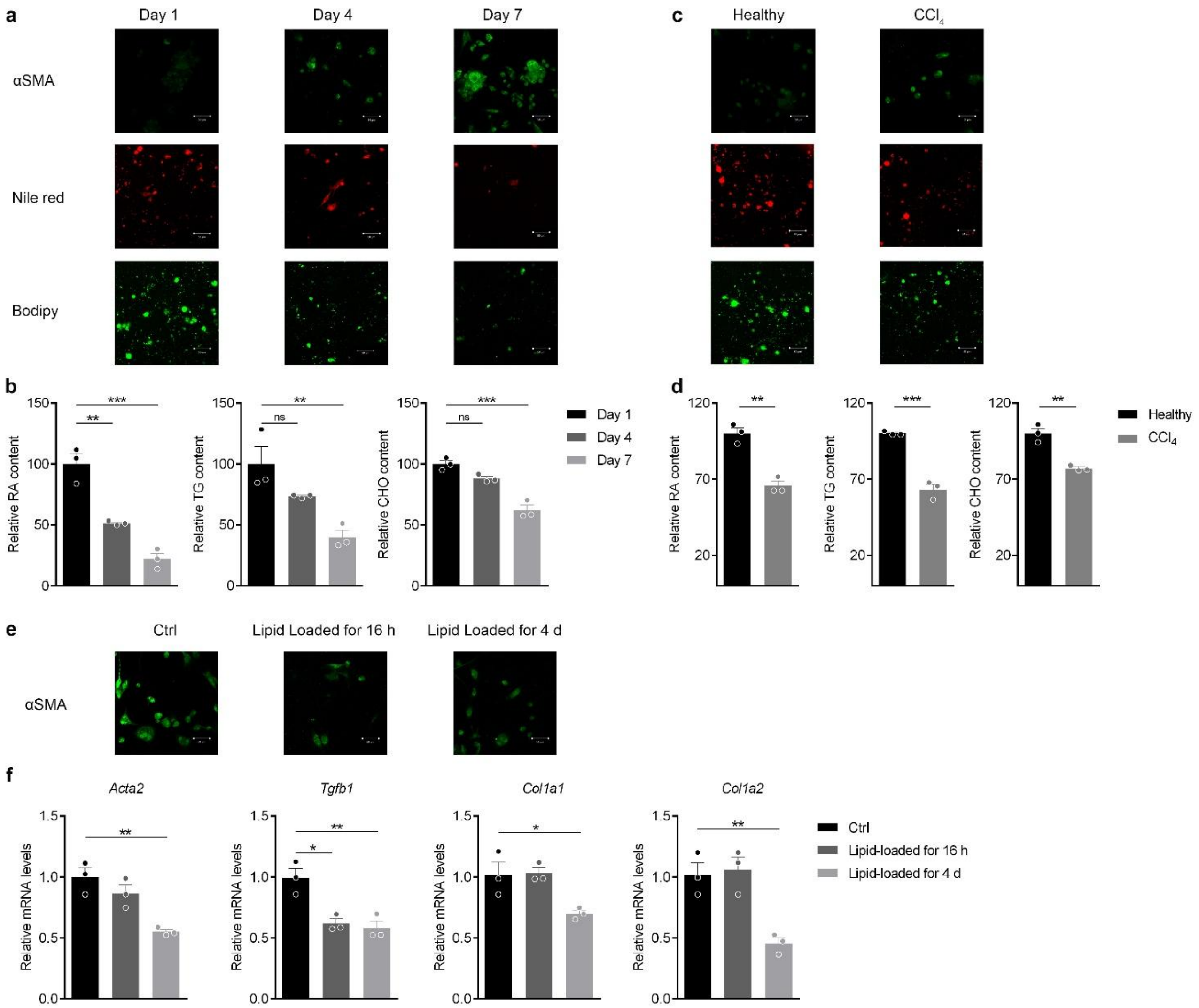

Supplementary Fig. 9. **LD loss controls the activation of HSCs**. **a-d**, Activation of HSCs paralleled with LD loss, n=3 biologically independent samples within these experiments. **a**, αSMA, Nile red and Bodipy staining of isolated primary HSCs cultured for variant days (representative of n=3. Scale bar, 50 μm.). **b**, Lipid quantitation analysis of isolated primary HSCs cultured for variant days. **c**, αSMA, Nile red and Bodipy staining of isolated primary HSCs from healthy or CCl_4-treated mice (representative of n=3. Scale bar, 50 μm.). **d**, Lipid quantitation analysis of isolated primary HSCs from healthy or CCl_4-treated mice. **e**,**f**, Lipid loaded HSCs showed repressed activation phenotype, n=3 biologically independent samples within these experiments. **e**, αSMA staining (representative of n=3. Scale bar, 50 μm.). **f**, Levels of pro-fibrotic gene mRNAs. Results are mean ± SEM, $*P< 0.05$, $**P< 0.01$, $***P< 0.001$, as assessed with Student's t-test or ANOVA.

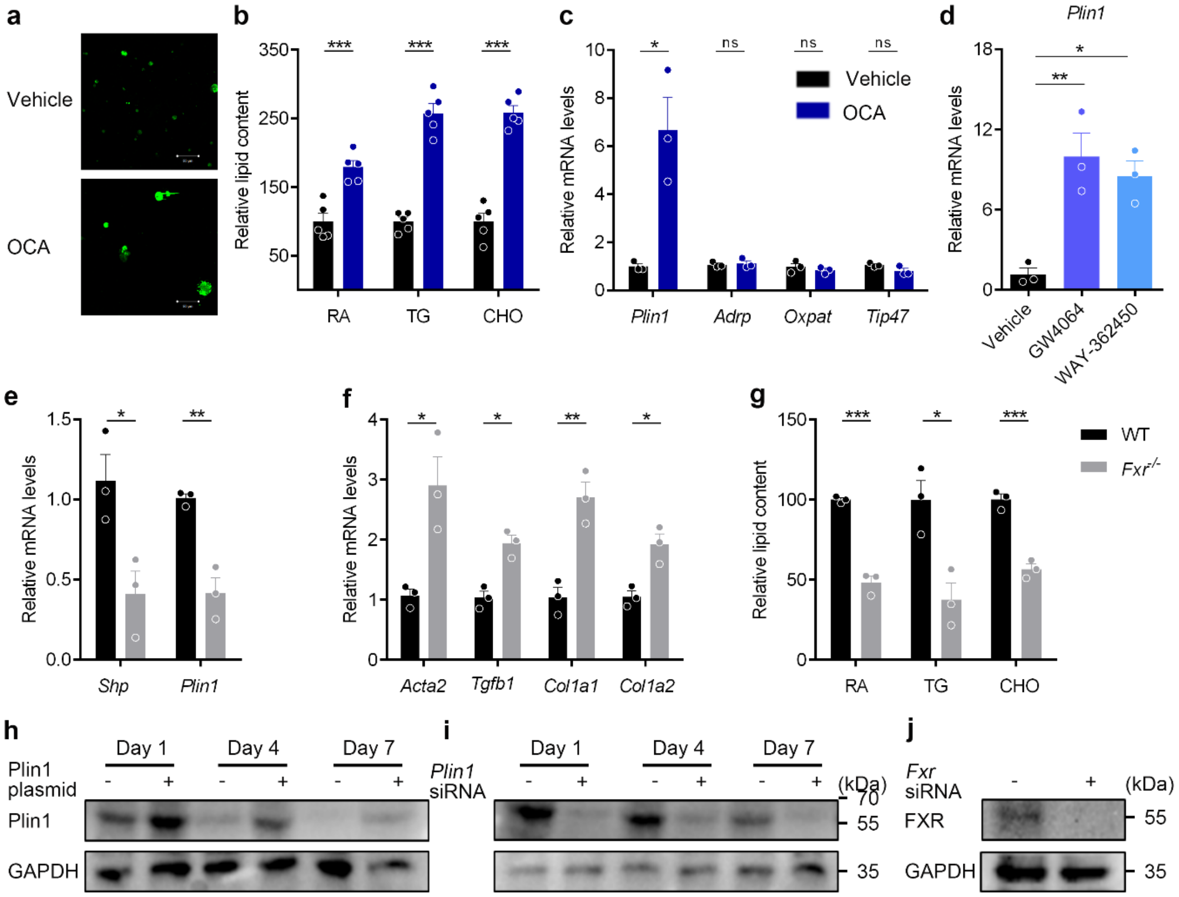

Supplementary Fig. 10. **Activation of FXR-Plin1 pathway prevents HSC activation and LD loss**. **a**, **b**, OCA treatment prevents LD loss. **a**, Bodipy staining of HSCs (representative of n=3 biologically independent samples. Scale bar, 50 µm.). **b**, Lipid quantitation analysis (n=5 biologically independent samples). **c**, Effect of OCA treatment on the expression of LD associated proteins (n=3 biologically independent samples within these experiments). **d**, Effect of FXR agonist on the mRNA expression of *Plin1*(n=3 biologically independent samples within these experiments). **e-g**, HSCs of *Fxr*$^{-/-}$ mouse shows suppressed expression of *Plin1* (**e**), enhanced expression of pro-fibrotic genes (**f**), as well as decreased lipid content (**g**), n=3 biologically independent samples within these experiments. h,i, Plin1 protein expression in primary HSCs transfected with Plin1 plasmid (h) or siRNA (i) and cultured for 1, 4, or 7 days. j, FXR protein expression in primary HSCs transfected with *Fxr* siRNA. Results are mean ± SEM, **P*< 0.05, ***P*< 0.01, and ns, statistically not significant, sas assessed with Student's t-test or ANOVA.

Supplementary table 1. Primer sequences for RT-PCR.

Genes	Sequences (5’-3’)	
Human *FXR*	Forward	GACTTTGGACCATGAAGACCAG
	Reverse	GCCCAGACGGAAGTTTCTTATT
Human *SHP*	Forward	AGGAATATGCCTGCCTGAAAGGG
	Reverse	CTGGTCGGAATGGACTTGAGGGT
Human *PLIN1*	Forward	ACATCAGTCTTGACAACTTGAGGA
	Reverse	GCACGGTGTATCGAGAGAGG
Human *GAPDH*	Forward	AATGAAGGGGTCATTGATGG
	Reverse	AAGGTGAAGGTCGGAGTCAA
Mouse *Fxr*	Forward	GCACGCTGATCAGACAGCTA
	Reverse	CAGGAGGGTCTGTTGGTCTG
Mouse *Shp*	Forward	GTACCTGAAGGGCACGATCC
	Reverse	GTGAAGTCTTGGAGCCCTGGT
Mouse *Acta2*	Forward	GCACCCAGCATGAAGATCAAG
	Reverse	TCTGCTGGAAGGTAGACAGCGAAG
Mouse *Col1a1*	Forward	TAGGCCATTGTGTATGCAGC
	Reverse	ACATGTTCAGCTTTGTGGACC
Mouse *Col1a2*	Forward	GCAGGGTTCCAACGATGTTG
	Reverse	GCAGCCATCGACTAGGACAGA
Mouse *Tgfb1*	Forward	AGGAGACGGAATACAGGGCT
	Reverse	CCACGTAGTAGACGATGGGC
Mouse *Plin1*	Forward	GGAGTGGCTGCAAGTGTTTC
	Reverse	ATCCCAGATTGCAGCAGACC
Mouse *Gapdh*	Forward	TTGATGGCAACAATCTCCAC
	Reverse	CGTCCCGTAGACAAAATGGT
Rat *Fxr*	Forward	TGGACTCATACAGCAAACAGAGA
	Reverse	GTCTGAAACCCTGGAAGTCTTTT

Rat *Shp*	Forward	CCTGGAGCAGCCCTCGT
	Reverse	AACACTGTATGCAAACCGAGGA
Rat *Acta2*	Forward	GCTCCATCCTGGCTTCTCTA
	Reverse	TAGAAGCATTTGCGGTGGAC
Rat *Col1a1*	Forward	AACCCCAAGGAGAAGAAGCA
	Reverse	AGCGTGCTGTAGGTGAATCG
Rat *Col1a2*	Forward	TCGCTCACAGCCTTCACTCAG
	Reverse	TGCGGGCAGGGTTCTTTCTA
Rat *Plin1*	Forward	GTGGCTCTCAGCTGCATGT
	Reverse	CTGGAAGCACTCACAGGTCC
Rat *Adrp*	Forward	AACCTGCCCTTGGTGAGCTC
	Reverse	GCCATTGACCACAGACTTGG
Rat *Tip47*	Forward	GTGGCTCACCTGGAATCAGAA
	Reverse	CTGAACACACTGAGTGCCTGG
Rat *Oxpat*	Forward	GGATGTCCGGTGATCAGAC
	Reverse	GTGCACGTGGCCCTGACCAG
Rat *Tgfb1*	Forward	TGCTTCAGCTCCACAGAGAA
	Reverse	TGGTTGTAGAGGGCAAGGAC
Rat *Gapdh*	Forward	AACGGCACAGTCAAGGCTGA
	Reverse	ACGCCAGTAGACTCCACGACAT
